# Combining PEGylated mito-atovaquone with MCT and Krebs cycle redox inhibitors as a potential strategy to abrogate tumor cell proliferation

**DOI:** 10.1038/s41598-022-08984-6

**Published:** 2022-03-24

**Authors:** Gang Cheng, Micael Hardy, Ming You, Balaraman Kalyanaraman

**Affiliations:** 1 Department of Biophysics, Medical College of Wisconsin, 8701 Watertown Plank Road, Milwaukee, WI 53226 USA; 2 Center for Disease Prevention Research, Medical College of Wisconsin, 8701 Watertown Plank Road, Milwaukee, WI 53226 USA; 3 Aix Marseille Univ, CNRS, ICR, UMR 7273, 13013 Marseille, France; 4 Center for Cancer Prevention, Houston Methodist Research Institute, 6670 Bertner Avenue, Houston, TX 77030 USA

**Keywords:** Drug discovery, Cancer prevention, NMR spectroscopy, Molecular biology

## Abstract

Glycolytic and mitochondrial oxidative metabolism, which are two major energy sources in tumors, are potential targets in cancer treatment. Metabolic reprogramming from glycolysis to mitochondrial oxidative metabolism and vice versa is an adaptive strategy with which tumor cells obtain energy to survive and thrive under the compromised conditions of glycolysis and mitochondrial respiration. Developing highly potent, nontoxic, and tumor-selective oxidative phosphorylation (OXPHOS) inhibitors may help advance therapeutic targeting of mitochondrial drugs in cancer. The FDA-approved antimalarial drug atovaquone (ATO), a mitochondrial complex III inhibitor, was repurposed in cancer treatment. Here, we developed a new class of PEGylated mitochondria-targeted ATO (Mito-(PEG)n-ATO). Depending on the PEGylation chain length (n), Mito-PEG-ATO analogs inhibit both mitochondrial complex I- and complex III-induced oxygen consumption in human pancreatic (MiaPaCa-2) and brain (U87MG) cancer cells. Mito-PEG_5_-ATO is one of the most potent antiproliferative mitochondria-targeted compounds (IC_50_ = 38 nM) in MiaPaCa-2 cells, and is more effective than other inhibitors of OXPHOS in MiaPaCa-2 and U87MG cells. Furthermore, we show that the combined use of the most potent OXPHOS-targeted inhibitors (Mito-PEG_5_-ATO) and inhibitors of monocarboxylate transporters (MCT-1 and MCT-4), Krebs cycle redox metabolism, or glutaminolysis will synergistically abrogate tumor cell proliferation. Potential clinical benefits of these combinatorial therapies are discussed.

## Introduction

Metabolic reprogramming, from glycolysis to mitochondrial oxidative metabolism and vice versa, is a dynamic process and one of the hallmarks of tumorigenesis, tumor growth, progression, and metastasis^1^. Both pathways—glycolysis and mitochondrial oxidative metabolism—are critical sources of energy in tumor cells. Dual inhibition of glycolytic and mitochondrial oxidative metabolism using 2-deoxyglucose (2-DG) or mitochondria-targeted drugs (MTDs) such as Mito-Q and Mito-metformin synergistically inhibit cancer cell proliferation and growth^2,3^. However, the clinical application of this modality remains limited due to the toxicity and therapeutic inefficacy of 2-DG^4^. Metformin, IACS-010759, atovaquone (ATO), and phenformin—molecules inhibiting oxidative phosphorylation (OXPHOS)—are undergoing clinical trials, alone or in combination with other standard-of-care therapies in cancer treatment (NCT01101438, NCT03291938, NCT03568994, and NCT03026517)^5–8^. OXPHOS is emerging as a vulnerable target in cancer therapy^9–11^. Monocarboxylate transport (MCT) inhibitors that control lactate uptake and efflux in cancer cells inhibit glycolytic metabolism^12^. The selective inhibition of lactate transport by AZD-3965 presents a novel way of targeting the metabolic phenotype in tumors that preferentially express MCT-1. AZD-3965 increased intratumor lactate concentration and intracellular pH. Excess production of lactate is removed from cells via MCT. The directionality of transport by MCTs depends on lactate and proton concentration gradients^13^. AZD-3965, an MCT-1 inhibitor, is undergoing a Phase I/II clinical trial for cancer therapy^14^. AZD-3965 was shown to increase oxidative metabolism in tumor cells^15^. Metformin, a weak OXPHOS inhibitor, enhanced the antiproliferative potency of AZD-3965 in tumor cells^16^.

Redox inhibitors, *i.e.*, CPI-613, CB-839, and V-9302, of the Krebs cycle, or tricarboxylic acid cycle, are undergoing clinical trials for treatment of cancer^17,18^. CPI-613 is a non-redox active lipoate analog that inhibits two different redox enzymes (alpha-ketoglutarate dehydrogenase) in the Krebs cycle^19^. A new combinatorial therapy regimen consisting of FOLFIRINOX and CPI-613 is undergoing a Phase III clinical trial in patients with metastatic pancreatic cancer^20^. CB-839 (*i.e.*, Telaglenastat) inhibits mitochondrial glutamine metabolism to glutamate catalyzed by glutamine synthase-1, leading to an overall inhibition of NADPH and glutathione synthesis^21^. V-9302, a small-molecule inhibitor of glutamine transporter and glutamine uptake into cells, sensitized the antitumor effect of other monotherapeutic agents in multiple tumor cells^21,22^. Under conditions of hypoxia or energy stress, such as enhanced glycolysis and/or inhibition of mitochondria, glutamine serves as an alternate energy source. Several cancer cells, including pancreatic and brain cancer, are addicted to the glutamine for proliferation^23–25^.

**Figure 1:**
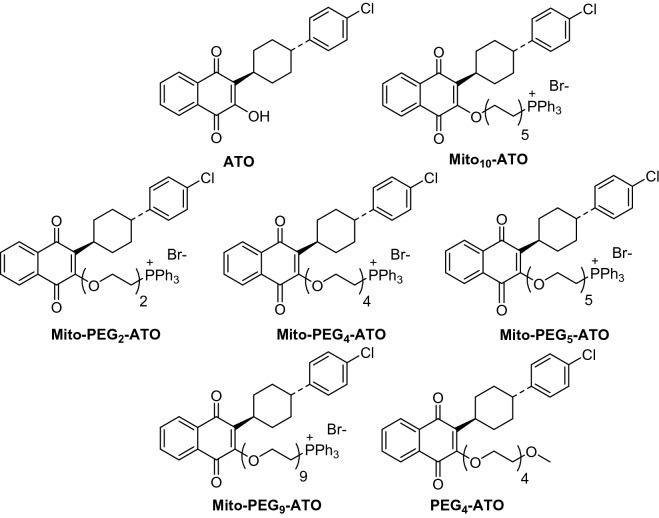
Chemical structures of Mito-PEG-ATO analogs and related compounds.

ATO, a hydroxy-1,4-naphthoquinone analog of ubiquinone (Q), also known as coenzyme Q10 (Fig. 1), is an FDA-approved antimicrobial drug used to treat malaria caused by the parasites *Pneumocystis jirovecii* and *Plasmodium falciparum*^26^. ATO is the first clinically approved drug that targets *Plasmodium cytochrome bc*_1_ complex in mitochondria^27^. Also, ATO acts as a competitive inhibitor of mitochondrial complex III by displacing ubiquinol at the active site of the cytochrome *bc*_1_ complex, inhibiting mitochondrial respiration and mitochondrial membrane potential in parasites and killing them^28^. Recently developed mitochondria-targeted analogs of ATO showed much greater antiproliferative potencies (> 100) compared with ATO^29^. A combinatorial therapy regimen consisting of either MCT inhibitors or Krebs cycle redox inhibitors and OXPHOS inhibitors will represent a new form of less toxic cancer therapy that differs mechanistically from standard cytotoxic therapies.

In this study, we discovered that PEGylation of mitochondria-targeted atovaquone (Mito-ATO) forms a new and very potent class of OXPHOS inhibitors and antiproliferative agents. PEGylation is the process of linking one or more polyethylene glycol (PEG) chains to a protein, peptide, or small molecule inhibitor of mitochondrial respiration^30,31^. PEGylation can improve a drug’s solubility and stability, increase serum half-life, and reduce immunogenicity without compromising activity^30,31^. The chemical structures of ATO, Mito-ATO, and PEGylated Mito-ATO (Mito-PEG-ATO) analogs; the structures of other metabolic inhibitors; and the nuclear magnetic resonance (NMR) spectra are shown in Figs. 1, S1, and S2. Depending on the chain length, at nanomolar concentrations, Mito-(PEG)n-ATO analogs selectively inhibit both complex I- and complex III-induced oxygen consumption in human pancreatic and brain cancer cells. Mito-PEG-ATO synergistically enhances the antiproliferative potencies of inhibitors of MCT and glutaminase enzymes and glutamine uptake in pancreatic and brain cancer cells. Potential translation of this modality to clinical cancer treatment is discussed.

## Results

### PEGylated Mito-ATO potently decreased proliferation and mitochondrial respiration in MiaPaCa-2 and U87MG cells

**Figure 2:**
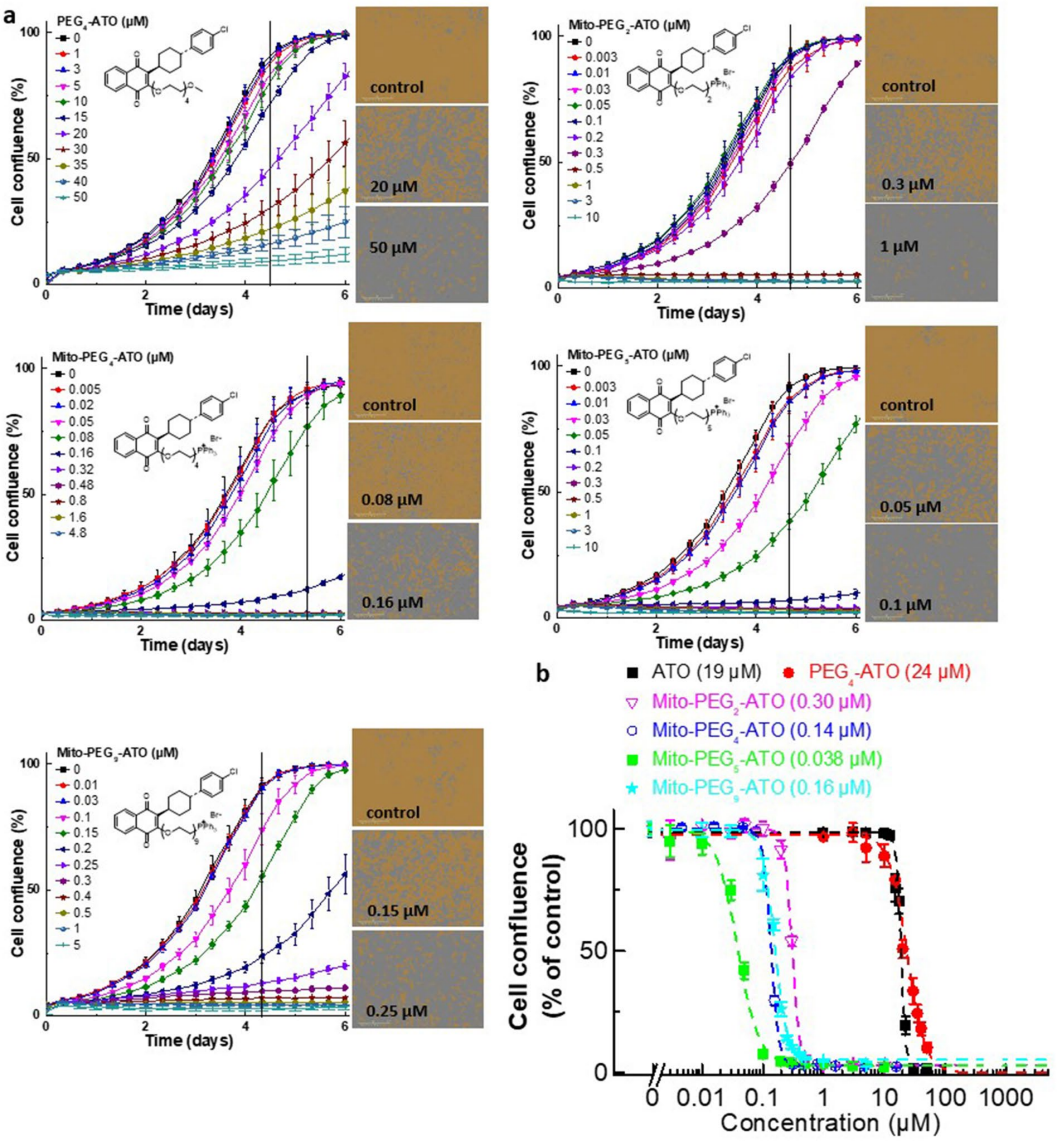
Effects of Mito-PEG-ATO analogs on the proliferation of human pancreatic cancer (MiaPaCa-2) cells. (**a**) The effects of Mito-PEG-ATO analogs on the proliferation of MiaPaCa-2 cells were monitored in the IncuCyte Live-Cell Analysis System. The IncuCyte analyzer provides real-time updates on cell confluence, based on segmentation of high definition-phase contrast images. Representative cell images were shown as a segmentation mask illustrated in brown when control cells reached 90% confluence (vertical solid black line). (**b**) The IC_50_ values were determined at the point at which control cells reached ~ 90% confluence (vertical solid black line). Relative cell confluence (control is taken as 100%) is plotted against concentration. Dashed lines represent the fitting curves used to determine the IC_50_ values as indicated. Data shown are the mean ± SD.

The antiproliferative effects of Mito-(PEG)n-ATO were investigated in human pancreatic cancer (MiaPaCa-2) cells and human brain cancer (U87MG) cells using an IncuCyte Live-Cell Analysis System (Essen Bioscience Inc., Ann Arbor, MI)^2,3^. Mito-PEG-ATO analogs (Mito-PEG_2_-ATO, Mito-PEG_4_-ATO, Mito-PEG_5_-ATO, and Mito-PEG_9_-ATO) dose-dependently inhibited proliferations of MiaPaCa-2 cells (Fig. 2a,b). Mito-PEG-ATO analogs exhibited a similar antiproliferative trend in U87MG cells with respect to variation in PEGylated side chain length in Mito-PEG-ATO (Fig. S4). The statistical analyses of cell proliferation data obtained at the half-maximal inhibitory concentration (IC_50_) (Fig. 2a) are shown in Table S1. The IC_50_ values of Mito-PEG_2_-ATO, Mito-PEG_4_-ATO, Mito-PEG_5_-ATO, and Mito-PEG_9_-ATO are 0.30 µM, 0.14 µM, 0.038 µM, and 0.16 µM, respectively (Figs. 2b, S5). Results indicate that the antiproliferative potency is dependent on the PEG chain length and that, after reaching a maximal potency for Mito-PEG_5_-ATO, it begins to decrease with further increase in PEG chain length (*e.g.*, Mito-PEG_9_-ATO). This is similar to that of non-PEGylated Mito-ATO analogs reported previously (Fig. S5)^31^. Note that the number of atoms present in the linker chain is 10 for Mito-ATO_10_ and is almost equivalent to Mito-PEG_4_-ATO, where the number of atoms in the linker side chain is 11 (Table 1). In control experiments, PEG_4_-ATO with a similar PEG chain length but lacking the triphenylphosphonium (TPP^+^) was considerably less effective (IC_50_ = 24 µM, Fig. 2). This result confirms the earlier conclusions that the TPP^+^ moiety in Mito-PEG-ATO is essential for its enhanced antiproliferative activity. We compared the potency of Mito-PEG-ATO analogs with other OXPHOS inhibitors (metformin and phenformin) that are currently undergoing clinical trials for treatment of patients with pancreatic and other cancers. Results obtained from testing the efficacy of these compounds to inhibit MiaPaCa-2 cells indicate that Mito-PEG-ATO (Mito-PEG_5_-ATO in particular) is orders of magnitude more potent (Fig. S6). Mito-PEG-ATO analogs did not induce nonspecific cellular toxicity, as revealed by the SYTOX Green assay shown in Fig. S14.

**Table 1:**
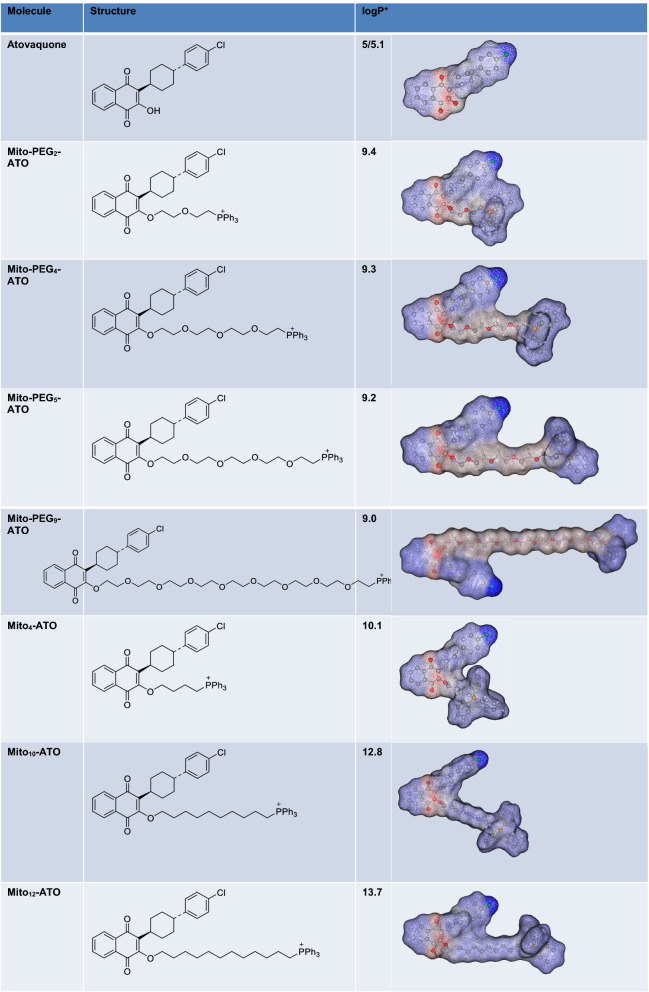
Calculated values of the octanol/water partition coefficients.

**Figure 3:**
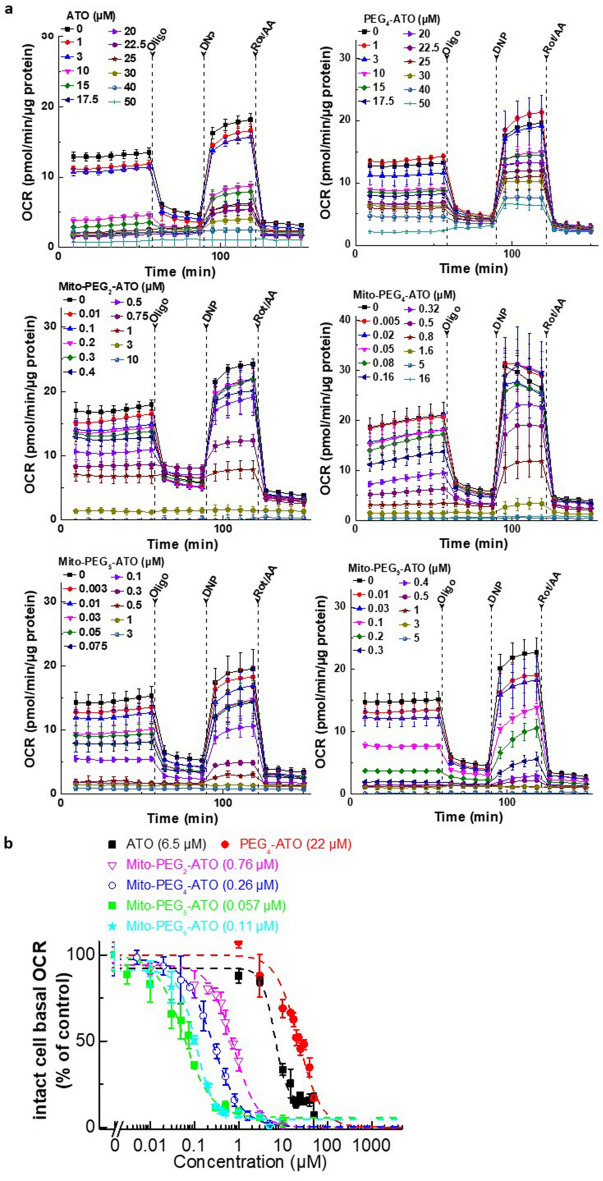
Effects of Mito-PEG-ATO analogs on intact cell mitochondria oxygen consumption of human pancreatic cancer (MiaPaCa-2) cells. (**a**) MiaPaCa-2 cells were treated with Mito-PEG-ATO analogs for 24 h, and concentration-dependent inhibition of mitochondrial respiration (OCR) in intact MiaPaCa-2 cells by Mito-PEG-ATO analogs was measured. (**b**) After eight baseline OCR measurements, the response to mitochondrial modulators (oligomycin, dinitrophenol, and rotenone/antimycin A as described in the Methods section) were recorded. The last three stable baseline OCR reading before oligomycin injection were plotted against the concentration of Mito-PEG-ATO analogs. Dashed lines represent the fitting curves used to determine the IC_50_ values as indicated. Data shown are the mean ± SD.

We also tested the relative efficacies of Mito-PEG_5_-ATO and ATO to inhibit the proliferation of human lung cancer (A549) cells and human colon cancer (HCT-116) cells (Fig. S7). The IC_50_ values of Mito-PEG_5_-ATO and ATO to inhibit A549 cell proliferation are 0.11 µM and 24 µM, respectively, and to inhibit HCT-116 cell proliferation are 0.14 µM and 20 µM, respectively. Mitochondrial respiration and complex activities were assessed using the Seahorse technique measuring the oxygen consumption rate (OCR)^32,33^. We first tested the efficacy of Mito-PEG-ATO analogs with varying PEG chain lengths on mitochondrial respiration in intact cells (Fig. 3). MiaPaCa-2 cells were treated with Mito-PEG-ATO analogs at different concentrations for 24 h, and the OCR was measured. Inhibition of basal mitochondrial respiration was, in part, linearly related to the increasing PEG chain length. As shown in Fig. 3, OCR inhibition was maximal at a PEG chain length of 5. The IC_50_ values (concentrations inhibiting 50% of OCR) for different Mito-PEG-ATO analogs are shown in Fig. 3b. Statistical analyses of the results for OCR in intact cells treated with Mito-PEG-ATO analogs at the IC_50_ are shown in Table S2. The OCR was suppressed in U87MG cells treated with Mito-PEG_4_-ATO (Fig. S8). Mito-PEG-ATO analogs, depending on their potency to inhibit OCR, enhanced the extracellular acidification rate (ECAR) as a compensatory increase in the glycolytic mechanism (Fig. S9). As shown, Mito-PEG_4_-ATO stimulated ECAR in MiaPaCa-2 cells at nanomolar levels and in U87MG cells (Fig. S10).

**Figure 4:**
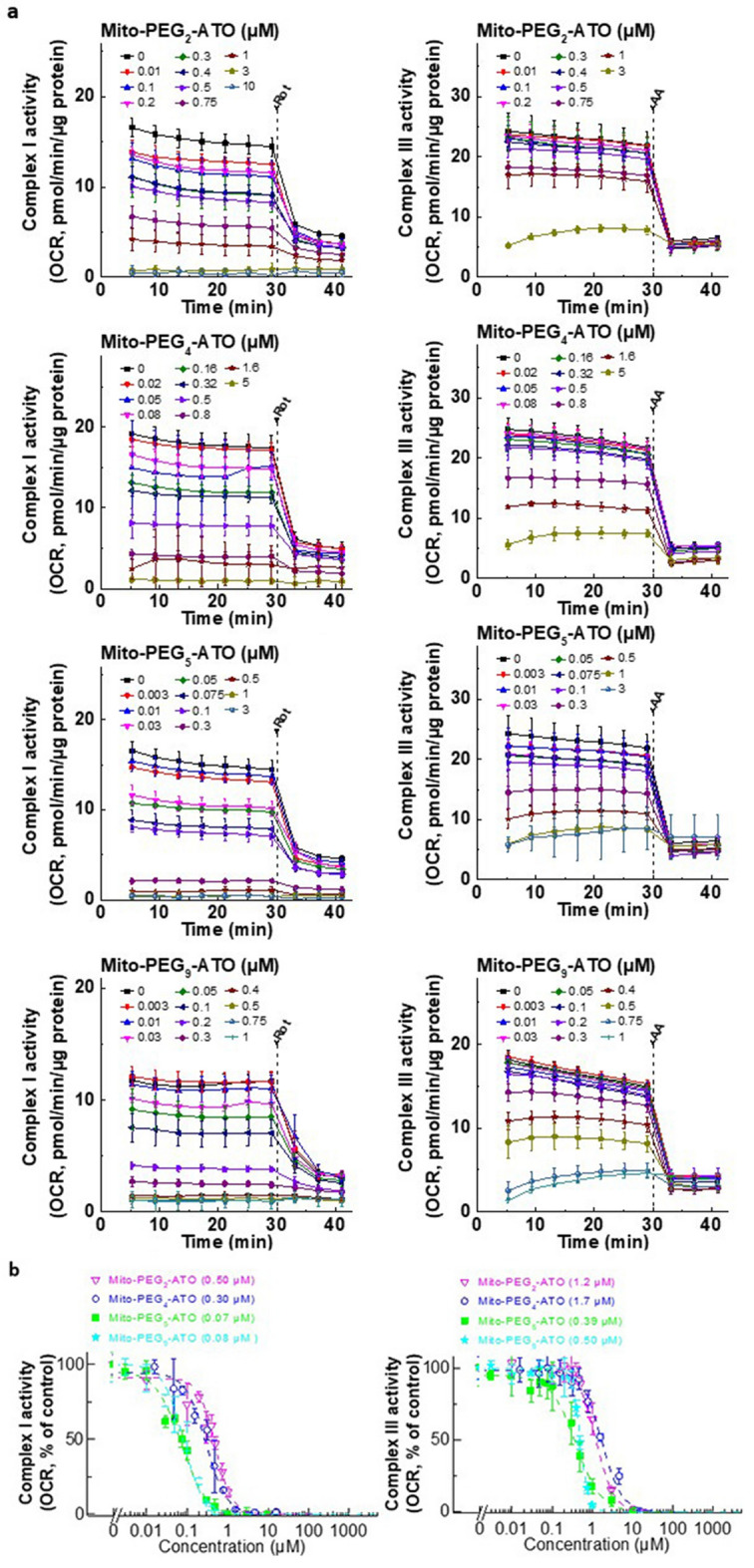
Effects of Mito-PEG-ATO analogs on the oxygen consumption by mitochondrial complexes I and III. (**a**) Dose-dependent effects of Mito-PEG-ATO analogs on complex I- and complex III-dependent oxygen consumption were measured in MiaPaCa-2 cells after the cells were treated with Mito-PEG-ATO analogs as indicated for 24 h. Mitochondrial complex I activity (left) and complex III activity (right) were monitored by a Seahorse XF-96 Extracellular Flux Analyzer. Either rotenone (complex I inhibitor) or antimycin A (complex III inhibitor) was acutely added and OCR assayed immediately. (**b**) The mitochondrial complex I (left) and III (right) dependent oxygen consumption (calculated as rotenone or antimycin A inhibitable OCR) are plotted against concentration of Mito-PEG-ATO analogs. Dashed lines represent the fitting curves used for determination of the IC_50_ values. Data shown are the mean ± SD.

Next, we determined the effect of Mito-PEG-ATO analogs on mitochondrial complex I- and complex III-induced oxygen consumption (Fig. 4). Mito-PEG-ATO analogs effectively inhibited both complex I- and complex III-induced oxygen consumption. Mito-PEG_5_-ATO inhibited complex I- and complex III-induced oxygen consumption more potently than any other mitochondria-targeted agent tested so far in our laboratory. The IC_50_ values for Mito-PEG_5_-ATO inhibiting complex I- and complex III-induced oxygen consumption are 0.07 µM and 0.39 µM, respectively (Fig. 4b). The inhibitory effect of Mito-PEG-ATO analogs was maximal at a PEG chain length of 5, and further increasing the PEG chain length (*e.g.*, Mito-PEG_9_-ATO) decreased the extent of complex I- and complex III-induced oxygen consumption. Statistical analyses of the results for complex I- and complex III-induced oxygen consumption as a function of different PEG chain lengths in Mito-(PEG)n-ATO at the time of the IC_50_ calculations are shown in Table S3. Comparing the results on cell proliferation and mitochondrial respiration, we conclude that the IC_50_s of Mito-PEG-ATO analogs to inhibit pancreatic cancer cell proliferation and mitochondrial respiration are very similar, and that mitochondrial respiration is key to cancer cell growth^34^. Mito-PEG_4_-ATO inhibited both complex I- and complex III-induced oxygen consumption in U87MG cells (Fig. S11).

### Synergistic interaction between Mito-PEG-ATO and MCT inhibitors (AZD-3965 and syrosingopine)

**Figure 5:**
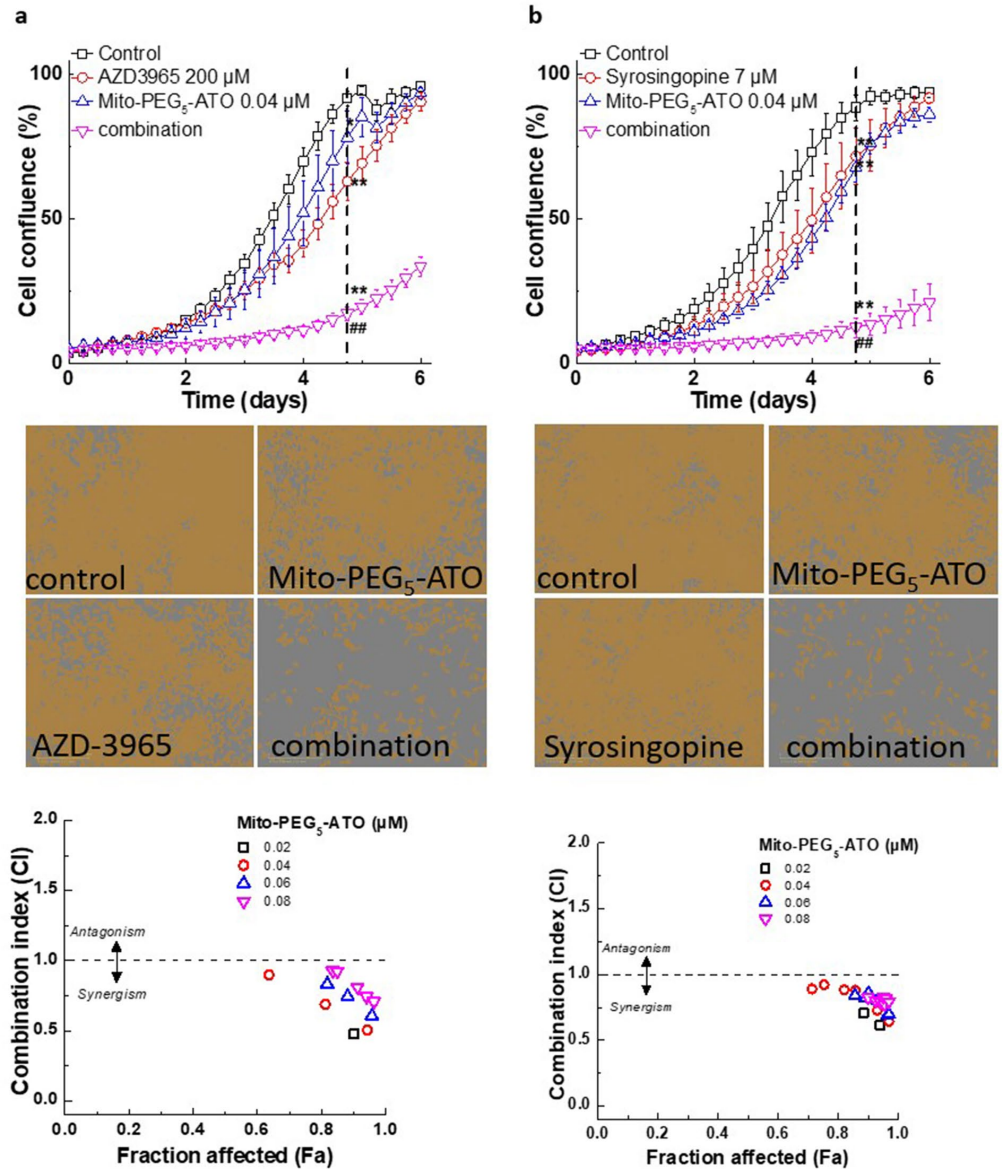
Synergistic inhibition of cell proliferation by Mito-PEG_5_-ATO and AZD-3965 or syrosingopine in human pancreatic cancer (MiaPaCa-2) cells. (Top) MiaPaCa-2 cells were treated with Mito-PEG_5_-ATO and AZD-3965 (**a**) or syrosingopine (**b**) independently and together at the indicated concentrations, and cell growth was monitored continuously. Data shown are the mean ± SD (n = 5). (Middle) Representative cell images are shown as a segmentation mask illustrated in brown when control cells reached ~ 90% confluence (vertical dashed line). **p < 0.01 vs control. ^##^*p* < 0.01 vs Mito-PEG_5_-ATO or AZD-3965 alone. (Bottom) Cell confluence (control cells reached ~ 90% confluence) is plotted against concentration for the synergistic calculation. Bottom panels (a, AZD-3965; b, syrosingopine) show the combination index–fraction affected plots. The fraction affected parameter is used as a measure of the efficiency of the drug(s), with a value of 0 indicating complete inhibition of cell confluence and a value of 1 indicating the lack of effect on cell confluence. Data shown are the mean ± SD.

Metformin (millimolar levels) and phenformin (high micromolar levels) in combination with AZD-3965 synergistically enhanced neuroblastoma cell death^15^. AZD-3965, an inhibitor of MCT-1, has been shown to increase the intracellular acidification in cancer cells due to accumulation of lactate and to inhibit glycolytic metabolism^35^. Because Mito-PEG_5_-ATO inhibited cell proliferation and mitochondrial respiration at nanomolar concentrations in MiaPaCa-2 cells, we tested the combination of Mito-PEG_5_-ATO and AZD-3965 using a concentration at which Mito-PEG_5_-ATO and AZD-3965 alone minimally affected cell proliferation (Fig. 5a); however, together they induced a dramatic decrease in MiaPaCa-2 cell proliferation. The combination index values show that the dual inhibition of cell proliferation is synergistic (Fig. 5a, bottom). We tested the synergistic effect of the combination of syrosingopine and Mito-PEG_5_-ATO (Fig. 5b). Figure S12 shows the potentiating effects of other mitochondrial inhibitors (metformin and phenformin) and AZD-3965 in MiaPaCa-2 cells. We also tested the synergistic effect of the combination of AZD-3965 and Mito-PEG_5_-ATO in glioblastoma cells, U87MG (Fig. S13).

The cell toxicity or cell death under these conditions was monitored using the SYTOX Green assay. All combinations (control, AZD-3965, Mito-PEG_5_-ATO, and AZD-3965 plus Mito-PEG_5_-ATO) yielded similarly low SYTOX Green counts (Fig. S14), indicating no increase in cell death even in the presence of a combination (e.g., AZD-3965 plus Mito-PEG_5_-ATO) that potently inhibited cell proliferation (Fig. 5a).

Intracellular adenosine triphosphate (ATP) levels were measured in MiaPaCa-2 cells treated with Mito-PEG_5_-ATO alone and in the presence of AZD-3965 and syrosingopine (Table S4). Results show that inhibition of MiaPaCa-2 cell proliferation in the presence of Mito-PEG_5_-ATO and MCT inhibitors is not due to intracellular ATP depletion.

### Synergistic interaction between Mito-PEG-ATO and Krebs redox metabolism and glutamine uptake inhibitors

**Figure 6:**
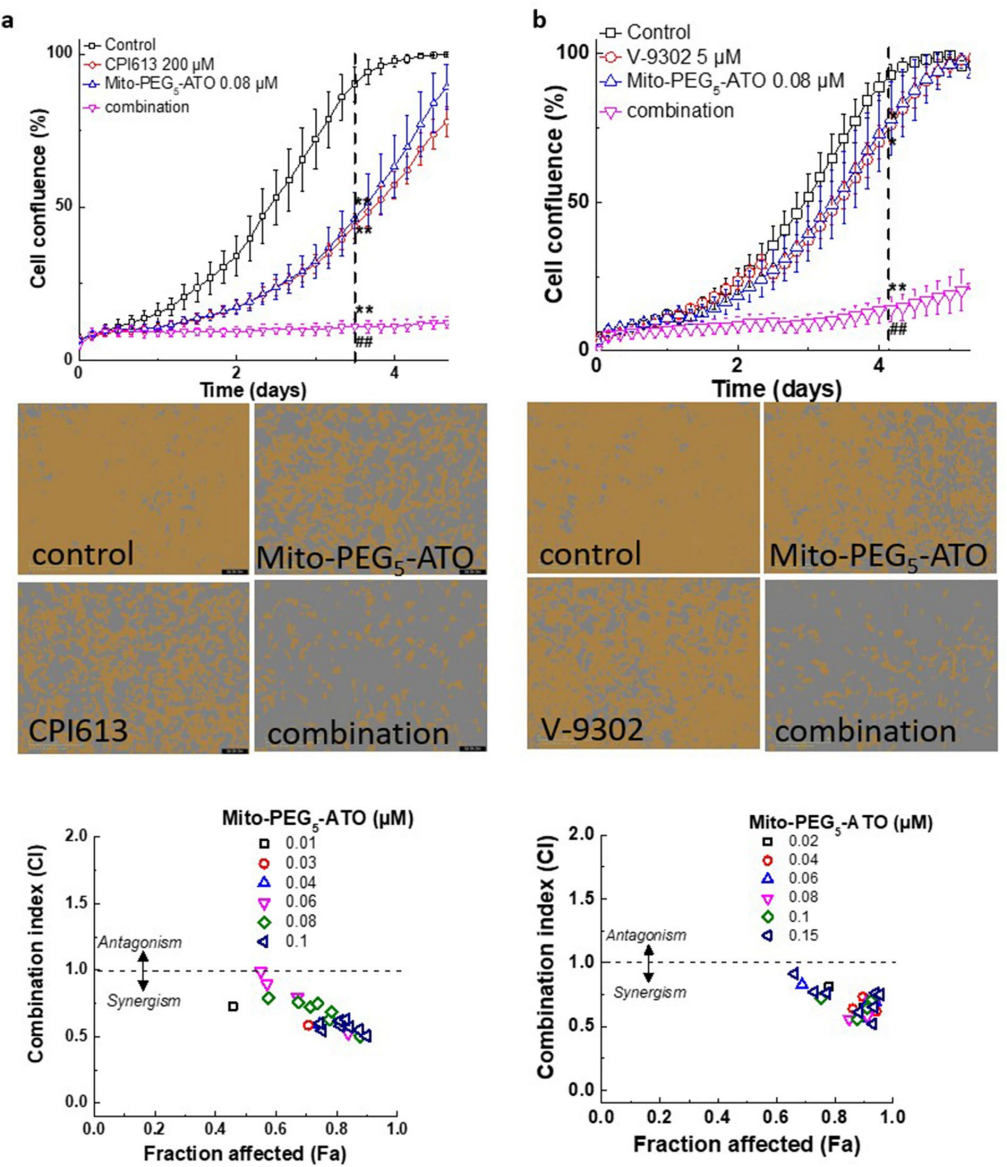
Synergistic inhibition of cell proliferation by Mito-PEG_5_-ATO and CPI-613 or V-9302 in MiaPaCa-2 cells. (Top) MiaPaCa-2 cells were treated with Mito-PEG_5_-ATO and CPI-613 (**a**) or V-9302 (**b**) independently and together at the indicated concentrations, and cell growth was monitored continuously. Data shown are the mean ± SD (n = 5). (Middle) Representative cell images are shown as a segmentation mask illustrated in brown when control cells reached ~ 90% confluence (vertical dashed line). **p < 0.01 vs control. ^##^*p* < 0.01 vs Mito-PEG_5_-ATO or AZD-3965 alone. **(Bottom)** Cell confluence (control cells reached ~ 90% confluence) is plotted against concentration for the synergistic calculation. Bottom panels (a, CPI-613; b, V-913) show the combination index–fraction affected plots. The fraction affected parameter is used as a measure of the efficiency of the drug(s), with a value of 0 indicating complete inhibition of cell confluence and a value of 1 indicating the lack of effect on cell confluence. Data shown are the mean ± SD.

**Figure 7:**
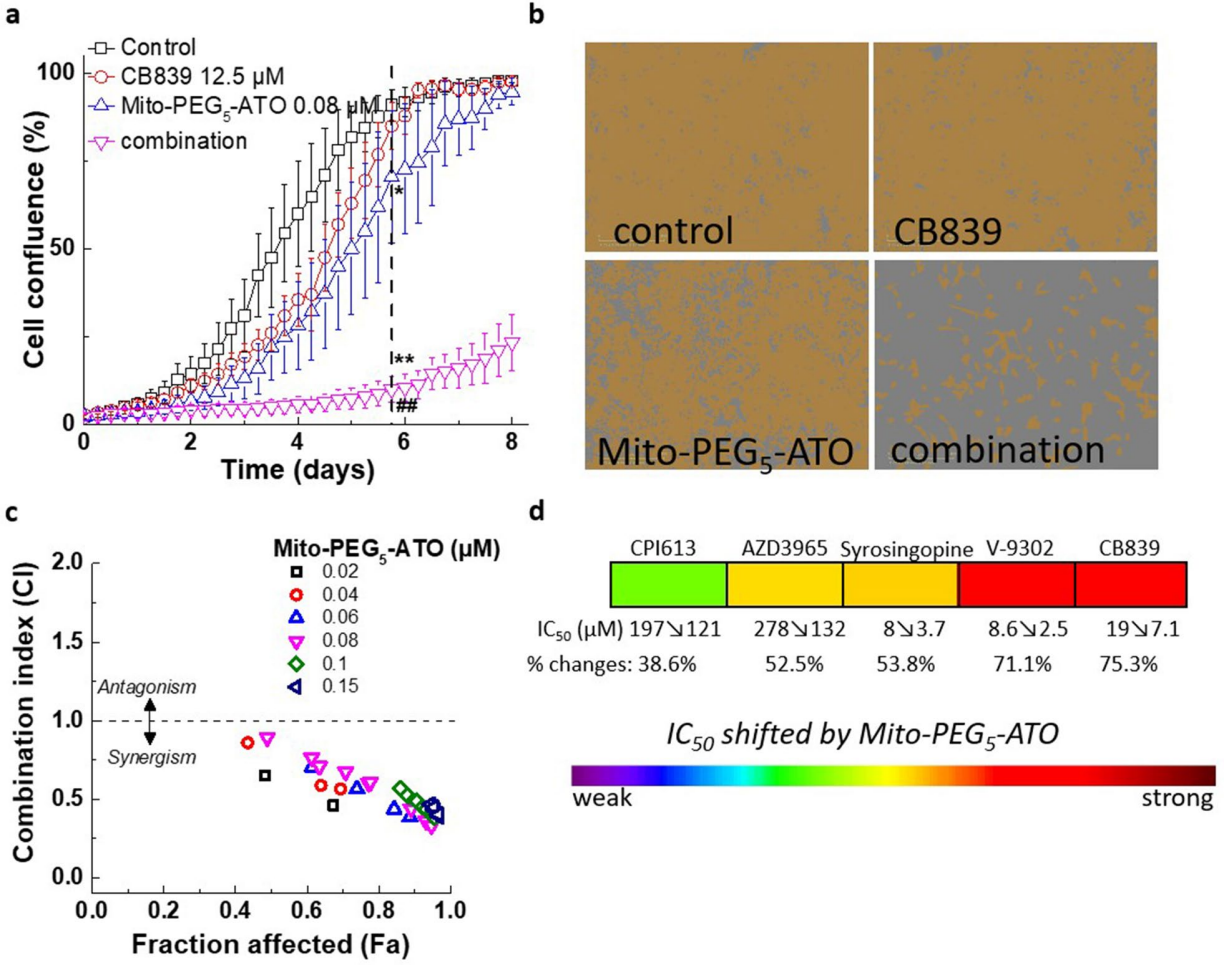
Synergistic inhibition of cell proliferation by Mito-PEG_5_-ATO and CB-839 in human pancreatic cancer (MiaPaCa-2) cells. (**a**) MiaPaCa-2 cells were treated with Mito-PEG_5_-ATO alone and in the presence of Mito-PEG_5_-ATO and CB-839 and cell growth was monitored continuously. (**b**) Representative cell images are shown as a segmentation mask illustrated in brown when control cells reached ~ 90% confluence (vertical dashed line). **p < 0.01 vs control. ^##^*p* < 0.01 vs Mito-PEG_5_-ATO or CB-839 alone. (**c**) Cell confluence (control cells reached ~ 90% confluence) is plotted against concentration for the synergistic calculation. (**d**) Heat map showing the enhanced effect of redox and MCT inhibitors in sensitizing the antiproliferative effect of Mito-PEG_5_-ATO. Deep red indicates strongest synergy (IC_50_ decreased from 19 µM to 7.1 µM, 75.3% inhibition) and deep green indicates weakest synergy (IC_50_ decreased from 121 µM to 91 µM, 38.6% inhibition).

MiaPaCa-2 cells were treated with redox metabolism inhibitors, with CPI-613 alone, and in combination with Mito-PEG-ATO (Fig. 6a). Even at high concentrations (200 µM), redox inhibitor CPI-613 only moderately inhibited cell proliferation. However, in combination with Mito-PEG_5_-ATO, CPI-613 synergistically inhibited cell proliferation (Fig. 6a, bottom). V-9302 is a competitive small-molecule antagonist that selectively targets the amino acid transporter ASCT2 and inhibits glutamine uptake into cells. Alone, neither V-9302 nor Mito-PEG_5_-ATO significantly inhibited cell proliferation (Fig. 6b), but in combination with Mito-PEG_5_-ATO it synergistically inhibited MiaPaCa-2 cell proliferation (Fig. 6b). Next, we tested the combined effect of Mito-PEG_5_-ATO and CB-839, another drug that inhibits mitochondrial glutamine metabolism (Fig. 7). The combination of Mito-PEG_5_-ATO and CB-839 exhibits synergy in inhibiting MiaPaCa-2 cell proliferation (Fig. 7a–c). These findings implicate a compensatory upregulation of glutamine metabolism in cells treated with Mito-PEG_5_-ATO. The heat map shows the relative strengths of synergy for Mito-PEG_5_-ATO in combination with redox and MCT inhibitors (Fig. 7d). Figure S15 shows the heat map for calculating the strength of synergy in different combinations (IC_50_s with or without Mito-PEG_5_-ATO) as has been previously reported^21^.

We measured both complex I- and complex III-induced oxygen consumption in MiaPaCa-2 cells in the presence of AZD-3965 or V-9302 with or without Mito-PEG_5_-ATO. Neither AZD-3965 nor V-9302 significantly affected the decrease in oxygen consumption induced by Mito-PEG_5_-ATO (Fig. S16). These results clearly indicate the synergistic inhibition of cell proliferation (Figs. 5, 6) observed in the presence of Mito-PEG_5_-ATO and AZD-3965 or V-9302 is not due to a synergistic decease in either complex I- or complex III-induced oxygen consumption.

### Intracellular ATP

Intracellular ATP levels were measured in MiaPaCa-2 cells treated with Mito-PEG_5_-ATO in the presence of redox metabolism inhibitors, AZD-3965 or syrosingopine (Table S4). The results indicate that these combinations, which effectively inhibit cell proliferation, are not due to intracellular ATP inhibition, as revealed by the intracellular ATP assay shown in Table S4.

### Analysis of intracellular oxidants: HE oxidation products

Cells were treated for 24 h with Mito-PEG_5_-ATO alone or in combination with AZD-3965 or CPI-613. 2-Hydroxyethidium (2-OH-E^+^) was used as a specific marker for superoxide, and diethidium (E^+^-E^+^) was used as a specific marker for intracellular one-electron oxidants^36,37^. The chemical structures of HE and oxidation/hydroxylation products are shown in Fig. S17. Mito-PEG_5_-ATO induced a slight but significant increase in 2-OH-E^+^, E^+^, and E^+^-E^+^ (Fig. S18). Figure S17b–d shows the relative concentrations of 2-OH-E^+^, E^+^, and E^+^-E^+^ in cells treated for 24 h with Mito-PEG_5_-ATO in combination with AZD-3965 (Fig. S17b), CPI-613 (Fig. S17c), or syrosingopine (Fig. S17d). Incubation for 48 h showed a similar trend (not shown). AZD-3965, syrosingopine, and CPI-613 only induced a modest and additive increase in intracellular oxidant-induced HE-derived products. It is likely that HE-derived oxidation products (E^+^ and E^+^-E^+^) result from mitochondrial iron or heme-mediated oxidation of HE. However, under these conditions, a highly synergistic inhibition in cell proliferation was observed (Figs. 5, 6). These results clearly do not support the tenet that superoxide or other one-electron oxidants are primarily responsible for the synergistic inhibition of cell proliferation observed in the presence of Mito-PEG_5_-ATO and AZD-3965 or CPI-613. N-acetylcysteine (10 mM) did not restore cell proliferation in cells treated with Mito-PEG_5_-ATO (not shown).

## Discussion

The most studied and widely used mitochondria-targeting vector is TPP^+^^38,39^. In molecules conjugated to TPP^+^, the positive charge is delocalized over the three phenyl rings. This lowers the activation energy barrier, allowing these molecules to penetrate the membrane and enter the mitochondria efficiently^40^. The hyperpolarized mitochondrial membrane of a cancer cell (− 220 mV) compared with that of a healthy mitochondrion (− 160 mV) facilitates the rapid and selective entry of molecules with a positive charge into mitochondrion^40^. The advantages of TPP^+^-based targeting molecules are the stability of TPP^+^ in the biological system, the low chemical reactivity toward cellular components, and the ability to modify the hydrophobicity by tethering alkylated linker side chains to various drugs^41^. The selective toxicity of TPP^+^-containing compounds in cancer cells as opposed to normal cells is its enhanced accumulation in tumor mitochondria, depending upon the alkyl side chain length^41^. However, with increasing alkyl side chain length, the hydrophobicity of the molecule is also increased (Table 1, Fig. 1). In contrast, PEGylation makes it possible to increase the side chain length without significantly increasing the hydrophobicity (Table 1). For example, the octanol/water partition coefficients (log P) values of Mito_10_-ATO (10 atoms in the linker side chain) and Mito-PEG_4_-ATO (11 atoms in the linker side chain) are 12.8 and 9.3, respectively (Table 1). Table 1 lists the calculated log P values along with the three-dimensional structures showing the relative hydrophilic and hydrophobic regions in Mito-PEG-ATO and Mito-ATO molecules. From the three-dimensional structures of the molecules (Table 1), it is evident that the PEGylated side chain region is more hydrophilic, enhancing the molecular hydrogen bonding interactions with water molecules and the hydrodynamic or steric volume.

PEGylated molecules exhibit several favorable pharmacological properties (*e.g.*, increased half-life and circulation time) and elicit improved pharmacodynamic and pharmacokinetic profiles^31,42^. Several PEGylated small molecules (*e.g.*, PEGylated curcumin) show increasing cancer therapeutic potential^43^. ATO has been shown to inhibit glioblastoma cell proliferation, and inhibition of STAT3 by ATO as a viable therapy for glioblastoma multiforme was proposed^44,45^. However, the ATO concentration in the brain was suggested to be too low to be chemotherapeutically effective. ATO and PEGylated drugs cross the blood–brain barrier. The PEG-coated carbon nanotube encapsulating mitochondria B cell lymphoma 2 inhibitor, ABT-737, was targeted to mitochondria of lung cancer cells^46^. Future studies will be focused on determining the in vivo pharmacokinetic profiles.

Previous studies using modeling and energy minimization showed that ATO inhibits complex III activity by docking into the Qo active site through hydrophobic and hydrogen bonding interactions with the Rieske protein and cytochromes^47,48^. Here, we report that tumor cell proliferation is greatly inhibited by attaching PEGylated TPP^+^ to ATO (Mito-PEG-ATO analogs). Depending on the length of the PEG chain attached to the TPP^+^ group, it is plausible that several hydrophobic/aromatic interactions between the ATO moiety and the amino acid residues within the binding site may stabilize the Mito-PEG-ATO analogs (*e.g.*, Mito-PEG_4_-ATO and Mito-PEG_5_-ATO) at the cytochrome *bc*_1_ pocket. Conjugating ATO to PEGylated TPP^+^ and increasing the PEGylation chain length influences their mitochondrial targeting and potency to inhibit oxygen consumption and cell proliferation. As shown in Figs. 2 and 3, and Tables S1 and S2, the IC_50_ value (0.038 µM) for Mito-PEG_5_-ATO to inhibit MiaPaCa-2 cell proliferation and oxygen consumption is much lower than the value (0.16 µM) obtained with Mito-PEG_9_-ATO. This trend is similar to that of Mito-ATO analogs with varying alkyl side chain lengths.

Several FDA-approved drugs (papaverine, ATO, metformin) that inhibit mitochondrial respiration are used as radiation sensitizers in radiation therapy of tumors and as enhancers of the efficacy of immunotherapy (*e.g.*, programmed cell death protein 1 blockade)^47,48^. Intracellular oxygen tension in tumors plays a critical role in the response to radiation therapy^49^. Enhanced tumor metabolism induces hypoxia, especially in solid tumors, and hypoxic tumor cells require a much higher dose of radiation for cell death as compared with normal cells^49–51^. The most effective approach to decrease tumor hypoxia (*i.e.*, to enhance oxygen concentration in tumors) is to decrease tumor oxygen consumption by inhibiting mitochondrial respiration^15^. Inhibition of mitochondrial respiration enhances radiation-induced pancreatic cancer cell killing, attributable to increased oxygen tension or decreased hypoxia. Results from this work suggest that Mito-PEG-ATO may be a more potent radiation sensitizer than other OXPHOS inhibitors that are undergoing clinical trials as radiation sensitizers. ATO, a complex III inhibitor, decreased hypoxia and downregulated hypoxia-regulated genes in non-small-cell lung cancer patients^52^. Targeted hypoxia-mitigating agents were shown to sensitize prostate cancer to immunotherapy^53^. PEGylated Mito-ATO analogs, some of the most potent and tumor cell selective mitochondrial complex I and III inhibitors, may serve as effective sensitizers of immunotherapy.

PEGylated OXPHOS inhibitors can greatly sensitize tumor cells and enhance the efficacy of MCT or redox inhibitors (Fig. 7). Previously, we showed that dual targeting of tumor cells with an OXPHOS complex I inhibitor and 2-DG, an inhibitor of glycolysis, synergistically enhanced their antiproliferative and antitumor effects in breast cancer cells^2^. This was one of the first reports on synergistic combinatorial antitumor effects of a TPP^+^-based OXPHOS inhibitor and an antiglycolytic inhibitor^2^. Targeting glutamine metabolism by inhibiting the glutaminase isoenzyme with CB-839 or with a novel inhibitor of glutamine transporter, V-9302, in tumors addicted to glutamine is showing promise in cancer treatment, but it is insufficient as a monotherapeutic agent^21^. However, Mito-PEG_5_-ATO greatly sensitized the antiproliferative effects of inhibitors of redox enzymes in the Krebs cycle (Fig. 7).

Lactate has emerged as more than just a byproduct, or the waste product, of the glycolytic metabolism in tumor cells^54^. AZD-3956 increased intracellular lactate and decreased extracellular lactate in tumor cells^15^. Depriving tumor cells of their ability to export lactate is potentially a promising therapeutic strategy. Glycolytic tumors express MCT-4 to excrete lactate to prevent intracellular over-acidification. Oxidative tumors overexpress MCT-1 to acquire more lactate for energy. An MCT inhibitor, AZD-3965, is being evaluated in clinical trials. As Mito-PEG-ATO enhances ECAR as a compensatory mechanism in tumor cells, AZD-3965 inhibits this pathway, leading to an overall synergistic inhibition of cell proliferation (Fig. 5). Lactate is a fuel in muscle and neurons. Although AZD-3965 is undergoing clinical trials as a promising antineoplastic agent, skeletal muscles and the brain also use lactate as an oxidative mitochondrial fuel and, consequently, MCT-1 inhibition by high concentrations of AZD-3965 could have deleterious side effects^55^. Combining AZD-3965 with potent OXPHOS inhibitors such as Mito-PEG_5_-ATO may decrease its toxic side effects.

CPI-613 and CB-839 are redox metabolism inhibitors targeted to the mitochondrial Krebs cycle. Both CPI-613 and CB-839 are in clinical trials for cancer treatment (NCT03374852 and NCT03875313)^56,57^. Mito-PEG-ATO analogs are targeted to mitochondrial OXPHOS. Results from this study show that targeting different oxidative metabolic sites in tumor mitochondria can synergistically inhibit tumor cell proliferation. Glutamine synthase, a key enzyme involved in glutamine synthesis, exhibits pro-tumorigenic characteristics, supporting nucleotide synthesis. More importantly, glutamine synthase is highly expressed in the tumor immune microenvironment and is a vulnerable therapeutic target in cancer treatment^58–60^. As shown in the heat map (Fig. 7d), Mito-PEG_5_-ATO can effectively enhance the therapeutic efficacy of redox inhibitors and MCT inhibitors.

Mito-ATO and other mitochondria-targeted compounds (Mito-hydroxyurea and Mito-magnolol) activate T cells and inhibit regulatory T cells (T_regs_) and myeloid-derived suppressor cells^29,61,62^. In addition to increasing intracellular lactate levels, AZD-3965 has pronounced effects on cancer cell metabolism (enhanced mitochondrial oxidative metabolism and improved bioenergetics). The combinatorial treatment with Mito-PEG-ATO could overwhelm this increase in mitochondrial oxidative metabolism and induce synergistic inhibition. This is important in the tumor immune microenvironment as inhibition of extracellular lactate enhanced the immune cell infiltration. MTDs could be used in conjunction with glutamine antagonizing drugs (*e.g.*, JHU-083) to decrease their systemic toxicity and synergistically enhance tumor suppression and antitumor T cell function^63^. Targeted blockade of glycolytic, glutaminolytic, and mitochondrial bioenergetic pathways remains, in theory, a promising approach in cancer treatment^1,2,11,63^. The present work reveals that PEGylation of MTDs further enhances their potency to nanomolar levels and, when combined with MCT inhibitors or Krebs cycle redox inhibitors, synergistic inhibition of tumor proliferation was possible. Potent OXPHOS inhibitors could be used to target cancer subtypes overexpressing OXPHOS genes. Enhancing the potency of OXPHOS inhibitors and therapeutic targeting through PEGylation is likely to decrease their off-target effects in immune cells (effector T cells and macrophages), thereby increasing tumor immunity.

Despite the many strengths of this study, we also recognize several limitations: Most PEGylated drugs have shown better pharmacokinetics. Previous reports indicate that targeting mitochondrial OXPHOS inhibits proliferation and growth in in vitro and in vivo models for several cancers^2,3,32,36^. Although the present data were obtained from in vitro cell culture experiments, the proposed strategy should be translatable to in vivo mouse xenografts. A related non-PEGylated analog, Mito-ATO, inhibited immunosuppressive T_regs_ and myeloid-derived suppressor cells in bone marrow cells and another mitochondria-compound-inhibited tumor growth in immune competent mice xenografts^29,61,62^. PEGylated biopharmaceuticals, micelles, nanoparticles, and liposomes were shown to induce immunogenicity^64^. Future studies should focus on the effect of Mito-PEG_5_-ATO in immune cells and immune competent mice tumor xenografts.

In summary, PEGylation of Mito-ATO generated a new class of very potent OXPHOS inhibitors, Mito-(PEG)n-ATO (n = 2–9), in tumor cells. The PEGylated molecules effectively inhibit both complex I- and complex III-induced oxygen consumption in human pancreatic (MiaPaCa-2) and brain (U87MG) cancer cells. Antiproliferative selectivity in tumor cells results from inhibition of OXPHOS in tumor mitochondria. PEGylation chain length influences their mitochondrial effects and antiproliferative potencies. PEGylated Mito-ATO (Mito-PEG_5_-ATO) is more potent and less cytotoxic. Mito-(PEG)n-ATO is not cytotoxic under conditions inhibiting cell proliferation. Mito-PEG_5_-ATO synergistically enhances the potency of MCT inhibitors and Krebs cycle redox metabolism inhibitors in tumor cells. Synergistic inhibition in cell proliferation observed in the presence of Mito-PEG_5_-ATO and MCT or redox inhibitors is probably not related to the slight increase in superoxide and oxidant formation induced by Mito-PEG_5_-ATO. The exact mechanism of synergy still remains to be established.

## Methods

### Synthesis of Mito-PEG-ATO analogs

The PEGylated mitochondria-targeted analogs of ATO (Mito-(PEG)n-ATO) were prepared in two steps, by reacting the appropriate PEGylated dibromoalcane with ATO in the presence of potassium carbonate in dimethyl formamide (DMF). The addition of triphenylphosphine on the bromoPEGylated ATO (PEG_n_-Br-ATO) led to the Mito-(PEG)n-ATO. In addition, the PEGylated ATO (PEG-ATO) was prepared using a similar procedure in the presence of bromo-2,5,8,11-tetraoxatridecane.

Synthesis of Mito-(PEG)n-ATO and PEG-ATO is presented in Fig. S3.

#### General

All chemicals and organic solvents were commercially available and were used as supplied. The reactions were monitored by thin layer chromatography (TLC) using Merck silica gel 60 F_254_. Crude materials were purified by flash chromatography on Merck silica gel 60 (0.040–0.063 mm). ^31^P NMR, ^1^H NMR and ^13^C NMR spectra, were recorded at 400.13 MHz spectrometers and 75.54 MHz, respectively. ^1^H NMR spectra were recorded using a Bruker DPX AVANCE 400 spectrometer (Marseille, PACA, France) equipped with a quattro nucleus probe. Chemical shifts (δ) are reported in ppm and *J* values in Hertz. ESI-HRMS (electrospray ionization high-resolution mass spectrometry) was performed on a SYNAPT G2 HDMS (Waters, Milford, MA).

NMR spectra are presented in Fig. S2.

#### Synthesis of Mito-PEG_2_-ATO, [2-(2-((3-(trans-4-(4-chlorophenyl)cyclohexyl)-1,4-dioxo-1,4-dihydronaphthalen-2-yl)oxy)ethoxy)ethyl]triphenylphosphonium bromide

To a mixture of ATO (0.5 g, 1.36 mmol) and potassium carbonate (0.26 g, 1.88 mmol) in DMF (5 mL) was added 1-bromo-2-[2-(2-bromoethoxy)ethoxy]ethane (0.63 g, 2.7 mmol). The mixture was stirred at 60 °C for 13 h. Then, dichloromethane (CH_2_Cl_2_) was added to the mixture as well as water (H_2_O) (20 mL). The organic layer was dried over sodium sulfate (Na_2_SO_4_). The solvent was removed under reduced pressure. Then, purification by flash chromatography (pentane/diethyl ether [Et_2_O] from 100% to 70/30) delivered the corresponding PEG_2_-Br-ATO (0.25 g, 35% yield).

High-resolution mass spectrometry (HRMS) calculated for PEG_2_-Br-ATO C_26_H_26_O_4_BrCl [M + H]^+^ 519.0756, found, 519.0758.

^1^H NMR (400.13 MHz, CDCl_3_), δ 8.08–7.99 (2H, m), 7.72–7.63 (2H, m), 7.27–7.22 (2H, m), 7.20–7.13 (2H, m), 4.56–4.50 (2H, m), 3.90–3.85 (2H, m), 3.84 (2H, t, *J* = 6.1), 3.42 (2H, t, *J* = 6.1), 3.28–3.18 (1H, m), 2.68–2.57 (1H, m), 2.27–2.174 (2H, m), 2.00–1.91 (2H, m), 1.78–1.68 (2H, m), 1.61–1.47 (2H, m).

A mixture of PEG_2_-Br-ATO (0.2 g, 0.36 mmol) and triphenylphosphine (93 mg, 0.36 mmol) in acetonitrile was stirred at reflux for 18 h. Purification by flash chromatography (CH_2_Cl_2_/ethanol [EtOH] 9/1) delivered the corresponding Mito-PEG_2_-ATO (0.2 g, 67% yield).

HRMS calculated for Mito-PEG_2_-ATO C_44_H_41_O_4_PCl^+^ [M]^+^ 699.2426, found, 699.2411.

^31^P NMR (400.13 MHz, CDCl_3_) δ 25.15. ^1^H NMR (400.13 MHz, CDCl_3_), δ 8.10–8.03 (1H, m), 8.01–7.94 (1H, m), 7.89–7.77 (6H, m), 7.77–7.64 (6H, m), 7.64–7.58 (5H, m), 7.28–7.22 (2H, m), 7.18–7.11 (2H, m), 4.28–4.19 (2H, m), 4.19–4.00 (4H, m), 3.69–3.57 (2H, m), 3.16–3.03 (1H, m), 2.63–2.48 (1H, m), 2.15–1.97 (2H, m), 1.91–1.80 (2H, m), 1.71–1.57 (2H, m), 1.57–1.40 (2H, m). ^13^C NMR (75 MHz, CDCl_3_) δ 185.2, 181.3, 157.4, 145.6, 139.1, 134.7, 134.6, 134.0, 133.9, 133.8, 133.2, 132.2, 131.5, 131.2, 130.1, 130.0, 128.4, 128.1, 126.4, 125.9, 119.2, 118.3, 71.7, 70.3, 64.3, 64.2, 43.2, 35.4, 34.4, 29.9, 25.5 (d, *J* = 52.1).

#### Synthesis of Mito-PEG_4_-ATO, [2-(2-(2-(2-((3-(trans-4-(4-chlorophenyl)cyclohexyl)-1,4-dioxo-1,4-dihydronaphthalen-2-yl)oxy)ethoxy)ethoxy)ethoxy)ethyl]triphenylphosphonium bromide

To a mixture of ATO (0.5 g, 1.36 mmol) and potassium carbonate (0.26 g, 1.88 mmol) in DMF (5 mL) was added 1,11-dibromo-3,6,9-trioxaundecane (1 g, 3.12 mmol). The mixture was stirred at 60 °C for 8 h. Then, CH_2_Cl_2_ was added to the mixture as well as H_2_O (20 mL). The organic layer was dried over Na_2_SO_4_. The solvent was removed under reduced pressure. Then, purification by flash chromatography (pentane/Et_2_O from 100% to 70/30) delivered the corresponding PEG_4_-Br-ATO (0.67 g, 81% yield).

HRMS calculated for PEG_4_-Br-ATO C_30_H_34_O_6_BrCl [M + NH_4_]^+^ 624.1547, found, 624.1540.

^1^H NMR (400.13 MHz, CDCl_3_), δ 8.08–8.03 (1H, m), 8.02–8.00 (1H, m), 7.73–7.63 (2H, m), 7.27–7.23 (2H, m), 7.20–7.15 (2H, m), 4.58–4.52 (2H, m), 3.88–3.83 (2H, m), 3.78–3.72 (2H, m), 3.70–3.65 (2H, m), 3.63–3.54 (6H, m), 3.45–3.39 (2H, m), 3.28–3.18 (1H, m), 2.68–2.57 (1H, m), 2.28–2.14 (2H, m), 2.00–1.92 (2H, m), 1.78–1.68 (2H, m), 1.61–1.47 (2H, m). ^13^C NMR (75 MHz, CDCl_3_) δ 185.3, 181.8, 157.7, 145.9, 138.3, 133.7, 133.0, 132.3, 131.4, 128.3, 128.1, 126.2, 125.8, 72.3, 71.1, 70.7, 70.6, 70.5, 70.4, 43.2, 35.3, 34.4, 30.2, 29.8.

A mixture of PEG_4_-Br-ATO (0.5 g, 0.82 mmol) and triphenylphosphine (0.24 g, 0.91 mmol) in acetonitrile was stirred at reflux for 18 h. Purification by flash chromatography (CH_2_Cl_2_/EtOH 9/1) delivered the corresponding Mito-PEG_4_-ATO (0.4 g, 56% yield).

HRMS calculated for Mito-PEG_4_-ATO C_48_H_49_BrClO_6_P^+^ [M]^+^ 787.2950, found, 787.2942.

^31^P NMR (400.13 MHz, CDCl_3_) δ 25.59. ^1^H NMR (400.13 MHz, CDCl_3_), δ 8.10–8.09 (1H, m), 8.02–8.00 (1H, m), 7.91–7.82 (6H, m), 7.78–7.69 (5H, m), 7.69–7.62 (6H, m), 7.30–7.29 (1H, m), 7.27–7.26 (1H, m), 7.20–7.15 (2H, m), 4.56–4.50 (2H, m), 4.29–4.19 (2H, m), 4.01–3.89 (m, 2H), 3.87–3.80 (2H, m), 3.63–3.55 (2H, m), 3.42–3.26 (2H, m), 3.34–3.28 (2H, m), 3.28–3.20 (3H, m), 2.69–2.59 (1H, m), 2.29–2.26 (2H, m), 2.03–1.92 (2H, m), 1.79–1.70 (2H, m), 1.62–1.40 (2H, m). ^13^C NMR (75 MHz, CDCl_3_) δ 185.3, 181.7, 157.7, 145.9, 138.5, 134.6, 134.5, 134.1, 133.9, 133.8, 133.1, 132.3, 131.5, 131.3, 130.0, 129.9, 128.4, 128.1, 126.3, 125.8, 119.4, 118.5, 72.3, 70.6, 70.5, 70.2, 70.1, 69.9, 64.0, 63.9, 43.2, 35.3, 34.5, 29.8, 25.3 (d, *J* = 52.1).

#### Synthesis of Mito-PEG_5_-ATO, [14-((3-(trans-4-(4-chlorophenyl)cyclohexyl)-1,4-dioxo-1,4-dihydronaphthalen-2-yl)oxy)-3,6,9,12-tetraoxatetradecyl]triphenylphosphonium bromide

To a mixture of ATO (0.5 g, 1.36 mmol) and potassium carbonate (0.26 g, 1.88 mmol) in DMF (5 mL) was added 1,14-dibromo-3,6,9,12-tetraoxatetradecane (1 g, 2.73 mmol). The mixture was stirred at 60 °C for 12 h. Then, CH_2_Cl_2_ was added to the mixture as well as H_2_O (20 mL). The organic layer was dried over Na_2_SO_4_. The solvent was removed under reduced pressure. Then, purification by flash chromatography (pentane/Et_2_O from 100% to 70/30) delivered the corresponding PEG_5_-Br-ATO (0.48 g, 54% yield).

HRMS calculated for PEG_5_-Br-ATO C_32_H_38_O_7_BrCl [M + NH_4_]^+^ 668.1810, found, 668.1804.

^1^H NMR (400.13 MHz, CDCl_3_), δ 8.05–8.00 (2H, m), 7.72–7.62 (2H, m), 7.27–7.22 (2H, m), 7.20–7.13 (2H, m), 4.56–4.50 (2H, m), 3.86–3.80 (2H, m), 3.76 (2H, t, *J* = 6.3), 3.69–3.52 (12H, m), 3.43 (2H, t, *J* = 6.2), 3.27–3.17 (1H, m), 2.66–2.56 (1H, m), 2.27–2.17 (2H, m), 2.00–1.88 (2H, m), 1.76–1.66 (2H, m), 1.60–1.46 (2H, m). ^13^C NMR (75 MHz, CDCl_3_) δ 185.3, 181.7, 157.6, 145.9, 138.4, 134.53, 134.50, 134.0, 133.9, 133.8, 130.0, 129.0, 128.4, 128.1, 126.3, 125.8, 119.4, 118.5, 72.3, 70.6, 70.5, 70.4, 70.19, 70.18, 69.8, 64.0, 63.9, 58.2, 43.2, 35.3, 34.5, 29.8.

A mixture of PEG_5_-Br-ATO (0.48 g, 0.74 mmol) and triphenylphosphine (0.22 g, 0.84 mmol) in acetonitrile was stirred at reflux for 24 h. Purification by flash chromatography (CH_2_Cl_2_ /EtOH 9/1) delivered the corresponding Mito-PEG_5_-ATO (0.35 g, 52% yield).

HRMS calculated for Mito-PEG_5_-ATO C_50_H_53_O_7_PCl^+^ [M]^+^ 831.3212, found, 831.3215.

^31^P NMR (400.13 MHz, CDCl_3_) δ 25.49. ^1^H NMR (400.13 MHz, CDCl_3_), δ 8.06–8.02 (1H, m), 7.99–7.96 (1H, m), 7.87–7.77 (6H, m), 7.76–7.65 (5H, m), 7.67–7.58 (6H, m), 7.25–7.19 (2H, m), 7.18–7.12 (2H, m), 4.54–4.45 (2H, m), 4.19–4.07 (2H, m), 3.95–3.84 (m, 2H), 3.84–3.79 (2H, m), 3.67–3.61 (2H, m), 3.57–3.50 (2H, m), 3.47–3.40 (2H, m), 3.37–3.26 (6H, m), 3.22–3.07 (1H, m), 2.67–2.53 (1H, m), 2.26–2.12 (2H, m), 1.99–1.90 (2H, m), 1.76–1.65 (2H, m), 1.60–1.44 (2H, m). ^13^C NMR (75 MHz, CDCl_3_) δ 185.3, 181.7, 157.6, 145.9, 138.4, 134.53, 134.50, 134.0, 133.9, 133.8, 133.1, 132.3, 131.4, 131.3, 130.0, 129.9, 128.4, 128.1, 126.3, 125.8, 119.4, 118.5, 72.3, 70.6, 70.5, 70.4, 70.19, 70.18, 69.8, 64.0, 63.9, 43.2, 35.3, 34.5, 29.8, 25.3 (d, *J* = 52.1).

#### Synthesis of Mito-PEG_9_-ATO, (26-((3-(trans-4-(4-chlorophenyl)cyclohexyl)-1,4-dioxo-1,4-dihydronaphthalen-2-yl)oxy)-3,6,9,12,15,18,21,24-octaoxahexacosyl)triphenylphosphonium bromide

Triphenylphosphine (6.3 g, 12 mmol) was dissolved in acetonitrile (35 mL) under argon. Bromine (3.8 g, 24 mmol) was added drop by drop at 0 °C. Then, 3, 6, 9, 12, 15, 18, 21, 24-octaoxahexacosane-1,26-diol (5.0 g, 12 mmol) was dissolved in acetonitrile (6 mL) and added dropwise. The reaction was stirred for 48 h at room temperature. The white residue was eliminated by filtration and the solvent was evaporated. The resulting orange residue was extracted several times with pentane. After evaporation of the combined extracts, the 1,26-dibromo-3, 6, 9, 12, 15, 18, 21, 24-octaoxahexacosane (Br-PEG_8_-Br) was obtained (4 g, 61%).

^1^H NMR (400.13 MHz, CDCl_3_), 3.83 (4H, t, *J* = 6.4 Hz), 3.72–3.65 (28H, m), 3.49 (4H, t, *J* = 6.4 Hz).

To a mixture of ATO (0.4 g, 1.1 mmol) and potassium carbonate (0.26 g, 1.9 mmol) in DMF (5 mL) was added Br-PEG_8_-Br (1.2 g, 2.2 mmol) in 3 mL of DMF. The mixture was stirred at 60 °C for 16 h. Then, CH_2_Cl_2_ was added to the mixture as well as H_2_O (20 mL). The organic layer was dried over Na_2_SO_4_. The solvent was removed under reduced pressure. Then, purification by flash chromatography (CH_2_Cl_2_ /Et_2_O, 50/50) delivered the corresponding PEG_9_-Br-ATO (0.80 g, 88% yield). The product was used without further purifications.

A mixture of PEG_9_-Br-ATO (0.8 g, 0.96 mmol) and triphenylphosphine (0.27 g, 1 mmol) in acetonitrile was stirred at reflux for 15 h. Et_2_O was added to precipitate the crude product. Purification by flash chromatography (CH_2_Cl_2_ /EtOH 95/05 to 90/10) delivered the corresponding Mito-PEG_9_-ATO (0.14 g, 14% yield).

HRMS calculated for Mito-PEG_9_-ATO C_58_H_69_O_11_PCl^+^ [M]^+^ 1007.4261, found, 1007.4261.

^31^P NMR (400.13 MHz, CDCl_3_) δ 25.65. ^1^H NMR (400.13 MHz, CDCl_3_), δ 8.10–7.99 (2H, m), 7.91–7.83 (6H, m), 7.79–7.73 (3H, m), 7.72–7.62 (8H, m), 7.30–7.28 (1H, m), 7.27–7.26 (1H, m), 7.21–7.15 (2H, m), 4.58–4.53 (2H, m), 4.27–4.19 (2H, m), 3.98 (1H, t, *J* = 5.6), 3.92 (1H, t, *J* = 5.6), 3.88–3.83 (2H, m), 3.70–3.66 (2H, m), 3.63–3.55 (18H, m), 3.54–3.50 (2H, m), 3.40–3.37 (2H, m), 3.36–3.32 (2H, m), 3.31–3.26 (2H, m), 3.25–3.19 (1H, m), 2.69–2.58 (1H, m), 2.29–2.15 (2H, m), 2.01–1.93 (2H, m), 1.79–1.70 (2H, m), 1.58–1.49 (2H, m). ^13^C NMR (75 MHz, CDCl_3_) δ 185.5, 181.8, 157.7, 146.0, 138.4, 135.0, 134.6, 134.2, 133.9, 133.8, 133.1, 132.8, 131.5, 131.4, 130.1, 130.0, 128.4, 128.2, 127.0, 126.3, 126.0, 125.9, 119.4, 118.7, 72.4, 70.7, 70.5, 70.3, 69.9, 64.2, 64.1, 43.3, 43.2, 35.3, 34.52, 34.48, 34.3, 29.8, 29.2, 25.5 (d, *J* = 52.4).

#### Synthesis of PEG_4_-ATO, 2-((2,5,8,11-tetraoxatridecan-13-yl)oxy)-3-(trans-4-(4-chlorophenyl)cyclohexyl)naphthalene-1,4-dione

To a mixture of ATO (0.3 g, 0.82 mmol) and potassium carbonate (0.11 g, 0.81 mmol) in DMF (3 mL) was added bromo-2,5,8,11-tetraoxatridecane (0.22 g, 0.82 mmol). The mixture was stirred at 60 °C for 12 h. Then, Et_2_O was added to the mixture as well as H_2_O (20 mL). The organic layer was washed twice with water and dried over Na_2_SO_4_. The solvent was removed under reduced pressure. Purification by flash chromatography (pentane/Et_2_O 80/20 to 100% Et_2_O) delivered the corresponding PEG-ATO (0.15 g, 33% yield).

HRMS calculated for PEG-ATO C_31_H_37_O_7_Cl [M + NH_4_]^+^ 574.2566, found, 574.2566.

^1^H NMR (400.13 MHz, CDCl_3_), δ 8.08–7.97 (2H, m), 7.72–7.62 (2H, m), 7.27–7.23 (2H, m), 7.20–7.15 (2H, m), 4.56–4.51 (2H, m), 3.87–3.82 (2H, m), 3.69–3.64 (2H, m), 3.61–3.56 (8H, m), 3.52–3.47 (2H, m), 3.34 (3H, s), 3.28–3.17 (1H, m), 2.66–2.56 (1H, m), 2.28–2.15 (2H, m), 2.00–1.90 (2H, m), 1.77–1.68 (2H, m), 1.61–1.47 (2H, m).

### Calculated values of the octanol/water partition coefficients

The calculated log P values of the Mito-PEG-ATO analogs were assessed using a QSAR (quantitative structure–activity relationship) analysis and rational drug design as a measure of molecular hydrophobicity (Table 1). This method also uses a consensus model built using the ChemAxon software (San Diego, CA)^65,66^. Table 1 lists the log P values along with the calculated regions of the relative hydrophilic and hydrophobic regions.

### Cell culture

The following cell lines were obtained from the American Type Culture Collection (Manassas, VA), where they were regularly authenticated: MiaPaCa-2 (ATCC Cat# CRL-1420, human pancreatic cancer cells), U87MG (ATCC Cat# HTB-14, human brain cancer cells), A549 (ATCC Cat# CCL-185, lung cancer cells), and HCT-116 (ATCC Cat# CCL-247, colon cancer cells). All cell lines were grown at 37 °C in 5% carbon dioxide. MiaPaCa-2 cells were maintained in Dulbecco's Modified Eagle Medium (Thermo Fisher Scientific, Waltham, MA; Cat# 11965) supplemented with 10% fetal bovine serum. U87MG, A549, and HCT-116 cells were maintained in RPMI 1640 medium (Thermo Fisher Scientific, Cat# 11875), supplemented with 10% fetal bovine serum. All cells were stored in liquid nitrogen and used within 20 passages after thawing.

### Cell proliferation measurements

The IncuCyte Live-Cell Analysis System was used to monitor cell proliferation^3,33,67^. As shown in previous publications^3,67^, this imaging system is probe-free and noninvasive, and enables continuous monitoring of cell confluence over several days. The increase in the percentage of cell confluence was used as a surrogate marker of cell proliferation. In a 96-well plate, cells were plated at 1,000 cells per well in triplicates and left to adhere overnight. Cells were then treated with Mito-PEG-ATO analogs, and the cell confluency was recorded over several days in the IncuCyte Live-Cell Analysis System.

### Mitochondrial oxygen consumption measurements

Mitochondrial oxygen consumption was measured using the Seahorse XF-96 Extracellular Flux Analyzer (Agilent, North Billerica, MA)^3,33,67,68^. To determine the intact cell mitochondrial function of MiaPaCa-2 cells in response to Mito-PEG-ATO analogs, we used the bioenergetic function assay previously described^2,3^. After cells were treated for 24 h, eight baseline OCR measurements were taken before injection of oligomycin (1 mg/mL) to inhibit ATP synthase, dinitrophenol (50 µM) to uncouple the mitochondria and yield maximal OCR, and rotenone (1 µM) and antimycin A (1 µM) to prevent mitochondrial oxygen consumption through inhibition of complexes I and III. From these measurements, indices of mitochondrial function were determined as previously described^2,3^. For mitochondrial complex activities assays, after cells were treated with Mito-PEG-ATO analogs for 24 h, the OCR-based assessment of mitochondrial complex activities was carried out on acutely permeabilized cells in the presence of different mitochondrial substrates, *i.e.*, pyruvate/malate for complex I and duroquinol for complex III^3,32,34,69^. Rotenone, malonate, and antimycin A (Sigma-Aldrich, St. Louis, MO) were used as specific inhibitors of mitochondrial complexes I, II, and III, respectively. Briefly, cells that were intact after treatments were immediately permeabilized using the Seahorse XF Plasma Membrane Permeabilizer (Agilent). The mitochondrial complex I-induced OCR was assayed in mannitol and sucrose buffer^69^ containing 10 mM pyruvate and 1.5 mM malate (substrates for complex I) and 10 mM malonate (which inhibits complex II activities). The mitochondrial complex III-driven OCR was assayed in a mannitol and sucrose buffer containing 0.5 mM duroquinol (substrate for complex III) as well as 1 µM rotenone and 10 mM malonate (which inhibit both complex I and II activities). The IC_50_ values were determined as previously reported^32^.

### Intracellular ATP measurements

After seeding cells overnight at 2 × 10^4^ per well in 96-well plates, cells were treated for 24 h. Intracellular ATP levels were determined in cell lysates using a luciferase-based assay per the manufacturer’s instructions (cat# FLAA, Sigma Aldrich, St. Louis, MO). In brief, the assay starts by rapidly adding an ATP assay mix solution (luciferase and luciferin solution, cat# FLAAM) to cell lysates. This is swirled briskly to mix, and the amount of light produced is immediately measured with a luminometer. The results were normalized to the total protein level measured in each well, as determined by the Bradford method (Bio-Rad Laboratories, Hercules, CA).

### Detection of HE oxidation products: Assessment of intracellular oxidants

Hydroethidine (HE) or dihydroethidium was used as a fluorogenic probe to assess the cellular generation of superoxide and other peroxidatic oxidants^36,37^. Cells were treated for 24 h with Mito-PEG_5_-ATO alone or in combination with AZD-3965 or CPI-613, followed by 1 h incubation with HE (10 µM). Cells were harvested and the cell pellet stored at − 80 °C until the day of high-performance liquid chromatography (HPLC) analysis. Sample preparation and HPLC analysis were performed as previously reported^32,36^. 2-OH-E^+^ was used as a specific marker for superoxide, and E^+^-E^+^ was used as a specific marker for intracellular one-electron oxidants^36,37^.

HE was purchased from Invitrogen (Carlsbad, CA). Stock solutions (20 mM and 5 mM, respectively) were prepared in deoxygenated DMSO (dimethyl sulfoxide) and stored at − 80 °C until use. Ethidium cation (bromide salt) was purchased from Sigma-Aldrich. The hydroxylated oxidation products from HE (2-OH-E^+^) were prepared by reacting the probes with Fremy’s salt as previously described in detail^70,71^. The dimeric products were prepared by reacting the probes with excess potassium ferricyanide, as detailed elsewhere^70^. Synthesized standards of all oxidation products of HE were purified by HPLC. HPLC-based measurements of HE and its oxidation products were performed using an Agilent 1100 system equipped with absorption and fluorescence detectors and refrigerated autosampler (4 °C). The samples (50 µL) were injected into a reverse phase column (Phenomenex, Kinetex C18, 100 mm × 4.6 mm, 2.6 µm) equilibrated with 20% acetonitrile and 80% water containing 0.1% trifluoroacetic acid. The compounds were eluted by increasing the content of acetonitrile from 20 to 56% over 4.5 min at a flow rate of 1.5 mL/min. The absorption detector was used to measure 3,8-diamino-6-phenylphenanthridine (at 290 nm; retention time: 2.5 min), HE-E^+^ (at 290 nm; retention time: 4.1 min), E^+^-E^+^ (at 290 nm; retention time: 4.3 min), and HE (at 370 nm; retention time: 1.6 min). A fluorescence detector (initial excitation at 358 nm and emission at 440 nm) was used to monitor HE (when present at low concentrations). At 2.3 min after injection, the fluorescence parameters were changed (excitation at 490 nm and emission at 567 nm) for sensitive detection of 2-OH-E^+^ (retention time: 3.2 min) and E^+^ (retention time: 3.3 min).

## Supplementary Information

Supplementary Information.
