# Supplementary material for: Combining PEGylated mito-atovaquone with MCT and Krebs cycle redox inhibitors as a potential strategy to abrogate tumor cell proliferation
Source: Sci Rep. 2022 Mar 24;12:5143. doi: 10.1038/s41598-022-08984-6 (PMC8948292; doi:10.1038/s41598-022-08984-6)
Supplement: Supplementary file 1 — Supplementary Information. [file 41598_2022_8984_MOESM1_ESM.docx]

**Combining PEGylated mito-atovaquone with MCT and Krebs cycle redox inhibitors as a potential strategy to abrogate tumor cell proliferation**

Gang Cheng,^1^ Micael Hardy,^3^ Ming You,^4^ Balaraman Kalyanaraman^1,2*^

^1^Department of Biophysics, ^2^Center for Disease Prevention Research, Medical College of Wisconsin, 8701 Watertown Plank Road, Milwaukee, WI 53226, United States

^3^Aix Marseille Univ, CNRS, ICR, UMR 7273, Marseille 13013, France

^4^Center for Cancer Prevention, Houston Methodist Research Institute, 6670 Bertner Avenue, Houston, TX 77030, United States

^*^Correspondence: balarama@mcw.edu

**Supplementary Materials**

**Figure S1. Chemical structures of** **MCT inhibitors, redox inhibitors, and glutamine inhibitors.**

**Figure S2. NMR Spectra.**

NMR Spectra of PEG_2_-Br-ATO

^1^H NMR (400.13 MHz, CDCl_3_)

NMR Spectra of Mito-PEG_2_-ATO

^13^P NMR (400.13 MHz, CDCl_3_)

^1^H NMR (400.13 MHz, CDCl_3_)

^13^CAPT (75 MHz, CDCl_3_)

NMR Spectra of PEG_4_-Br-ATO

^1^H NMR (400.13 MHz, CDCl_3_)

^13^CAPT (75 MHz, CDCl_3_)

NMR Spectra of Mito-PEG_4_-ATO

^13^P NMR (400.13 MHz, CDCl_3_)

^1^H NMR (400.13 MHz, CDCl_3_)

^13^CAPT (75 MHz, CDCl_3_)

NMR Spectra of PEG_5_-Br-ATO

^1^H NMR (400.13 MHz, CDCl_3_)

^13^CAPT (75 MHz, CDCl_3_)

NMR Spectra of Mito-PEG_5_-ATO

^13^P NMR (400.13 MHz, CDCl_3_)

NMR Spectra of Mito-PEG_5_-ATO

^1^H NMR (400.13 MHz, CDCl_3_)

^13^CAPT (75 MHz, CDCl_3_)

NMR Spectra of Br-PEG_8_-Br

^1^H NMR (400.13 MHz, CDCl_3_)

NMR Spectra of Mito-PEG_9_-ATO

^13^P NMR (400.13 MHz, CDCl_3_)

^1^H NMR (400.13 MHz, CDCl_3_)

^13^C (75 MHz, CDCl_3_)

NMR Spectrum PEG-ATO

^1^H NMR (400.13 MHz, CDCl_3_)

**Figure S3. Synthesis of Mito-(PEG)n-ATO and PEG-ATO.** Reagents and conditions: i, Br-PEG_n_-Br, K_2_CO_3_, DMF, 60°C, 8 h, 35–81%; ii, triphenylphosphine, CH_3_CN, reflux, 18 h, 14–67%; iii, Br-PEG_4_-Br, K_2_CO_3_, DMF, 60°C, 33%.

**
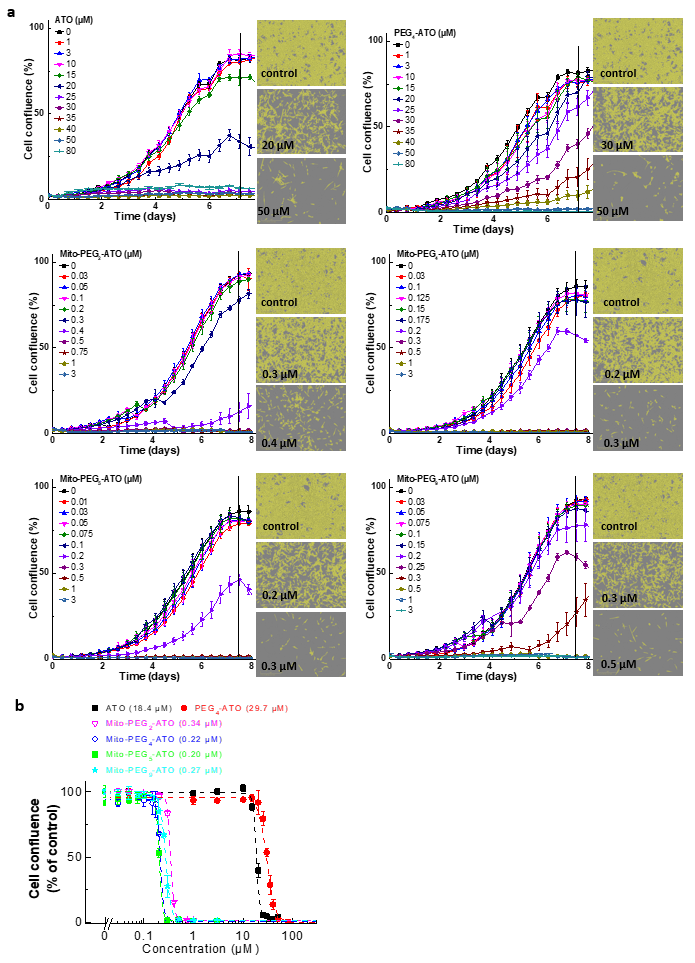
**

**Figure S4. Effects of Mito-PEG-ATO analogs on the proliferation of human brain cancer (U87MG) cells. (a)** Effects of Mito-PEG-ATO analogs on the proliferation of U87MG cells were monitored in the IncuCyte Live-Cell Analysis System. The IncuCyte analyzer provides real-time updates on cell confluence, based on segmentation of high definition-phase contrast images. Representative cell images were shown as a segmentation mask illustrated in brown when control cells reached 90% confluence (vertical solid black line). **(b)** The IC_50_ values were determined at the point at which control cells reached ~90% confluence (vertical solid black line in Panel a). Relative cell confluence (control is taken as 100%) is plotted against concentration. Dashed lines represent the fitting curves used to determine the IC_50_ values as indicated. Data shown are the mean±SD.

**
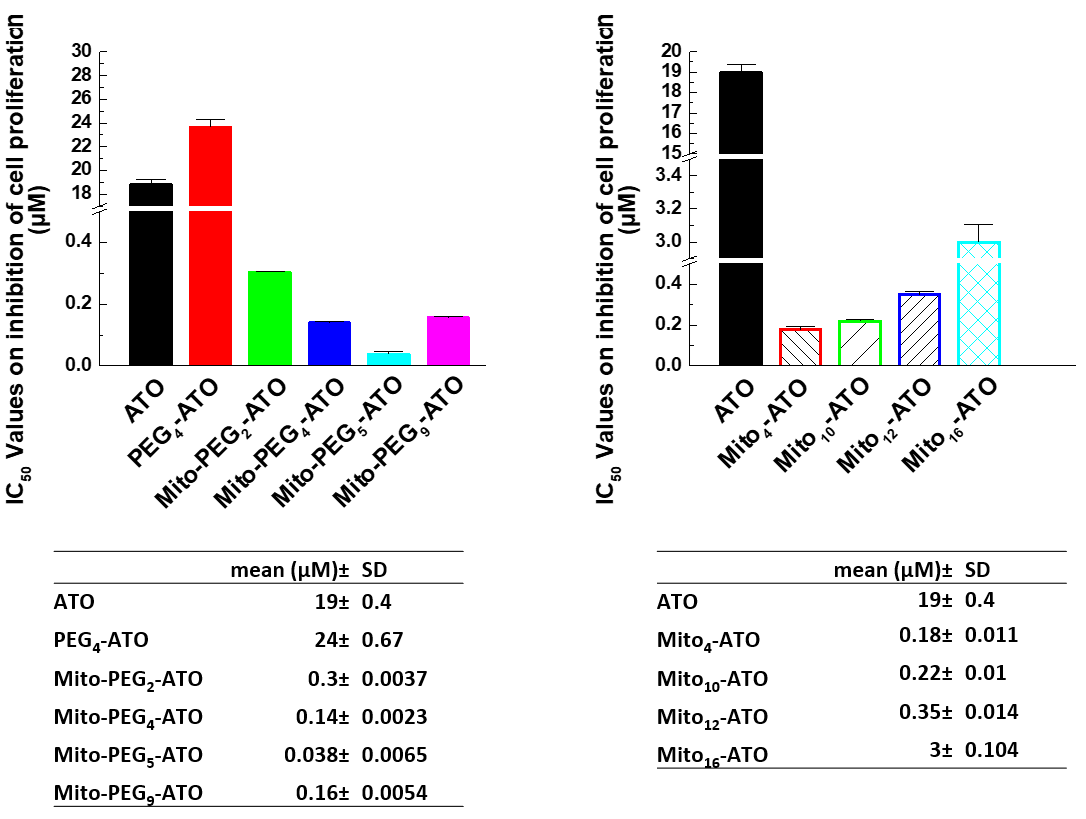
**

**Figure S5. Effects of Mito-PEG-ATO and Mito-ATO analogs on cell proliferation in human pancreatic cancer (MiaPaCa-2) cells.** Related to Figure 2.

**
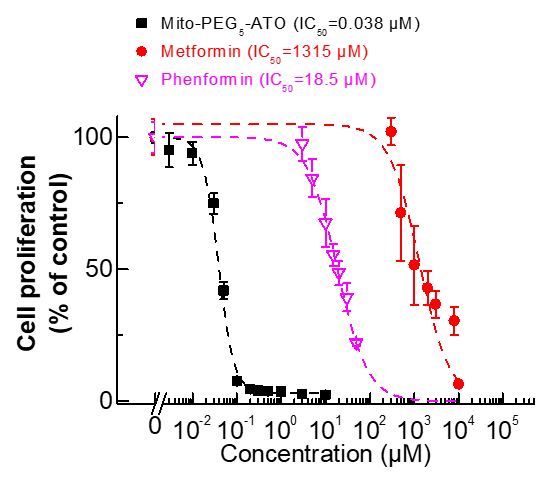
**

**Figure S6. Relative inhibitory effects of Mito-PEG_5_-ATO, metformin, and phenformin in human pancreatic cancer (MiaPaCa-2) cells.** The effects of Mito-PEG_5_-ATO, metformin, and phenformin on the proliferation of MiaPaCa-2 cells were monitored in the IncuCyte Live-Cell Analysis System. The IncuCyte analyzer provides real-time updates on cell confluence, based on segmentation of high-definition–phase contrast images. The IC_50_ values were determined at the point at which control cells reached ~90% confluence (vertical solid black line). Relative cell confluence (control is taken as 100%) is plotted against concentration. Dashed lines represent the fitting curves used to determine the IC_50_ values as indicated. Data shown are the mean±SD.

**
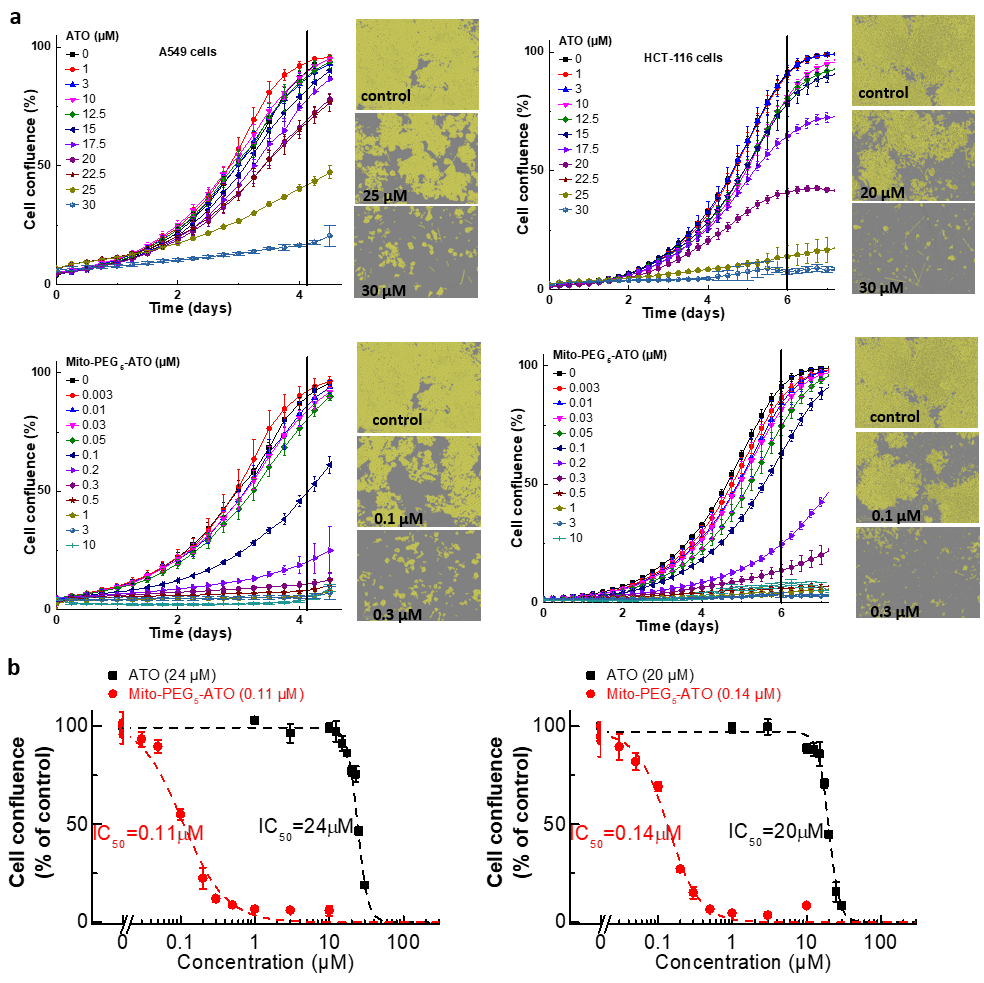
**

**Figure S7. Effects of Mito-PEG-ATO analogs on the proliferation of human lung cancer (A549) cells and human colon cancer (HCT-116) cells. (A)** The effects of Mito-PEG_5_-ATO and ATO on the proliferation of A549 *(left)* or HCT-116 *(right)* cells were monitored in the IncuCyte Live-Cell Analysis system. The IncuCyte analyzer provides real-time updates on cell confluence, based on segmentation of high definition-phase contrast images. Representative cell images were shown as a segmentation mask illustrated in brown when control cells reached 90% confluence (vertical solid black line). **(B)** The IC_50_ values for A549 *(left)* or HCT-116 *(right)* cells were determined at the point at which control cells reached ~90% confluence (vertical solid black line). Relative cell confluence (control is taken as 100%) is plotted against concentration. Dashed lines represent the fitting curves used to determine the IC_50_ values as indicated. Data shown are the mean±SD.

**
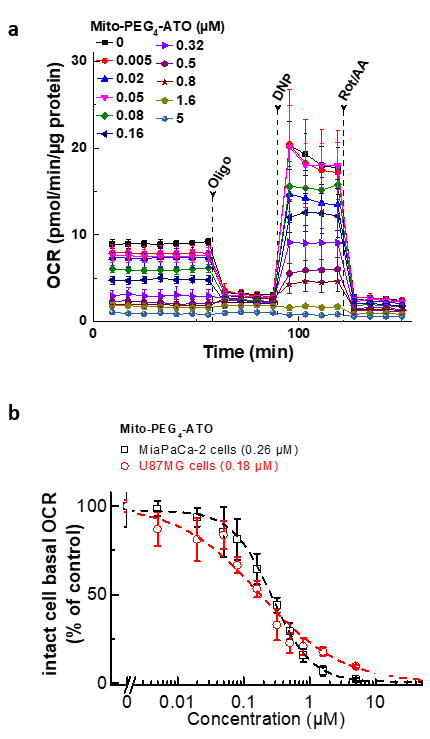
**

**Figure S8. Effects of Mito-PEG_4_-ATO on intact cell mitochondria oxygen consumption of human brain cancer (U87MG) cells. (a)** U87MG cells were treated with Mito-PEG_4_-ATO for 24 h and concentration-dependent inhibition of mitochondrial respiration (OCR) in intact U87MG cells by Mito-PEG_4_-ATO were measured. **(b)** After eight baseline OCR measurements, the response to mitochondrial modulators (oligomycin, dinitrophenol, and rotenone/antimycin A as described in the Methods section) were recorded. The last three stable baseline OCR reading before oligomycin injection was plotted against the concentration of Mito-PEG_4_-ATO (red open box). Dashed lines represent the fitting curves used to determine the IC_50_ values as indicated. Fitting curve for MiaPaCa-2 cells was from Fig. 3. Data shown are the mean±SD.

**
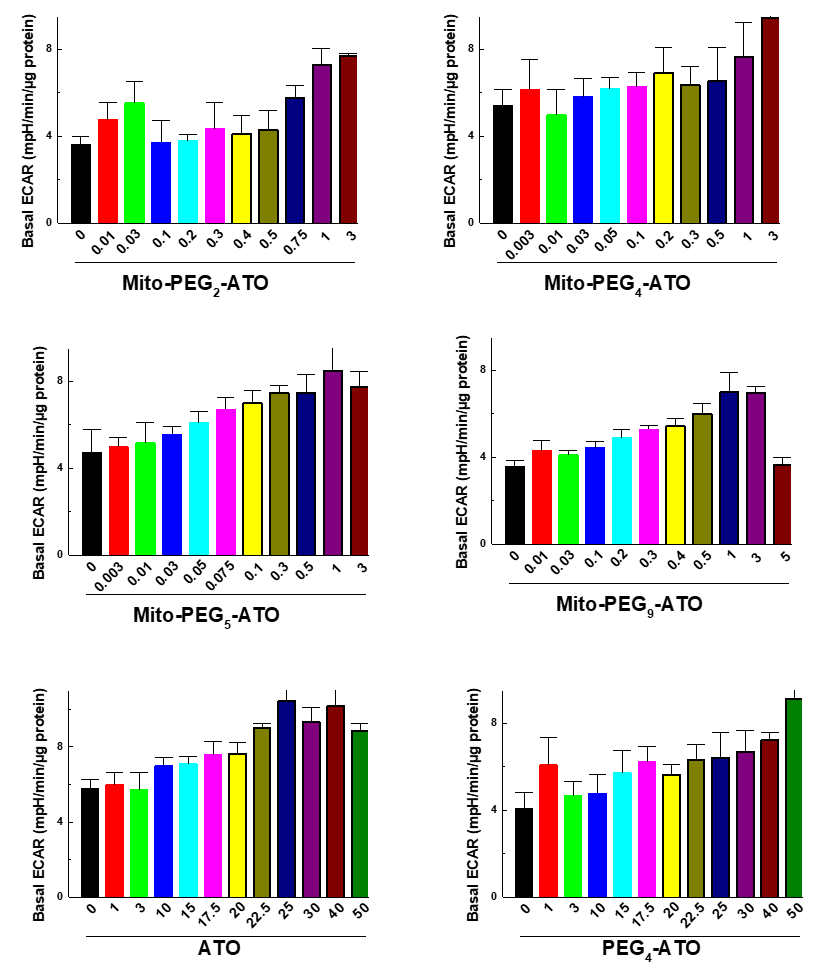
**

**Figure S9. Effects of Mito-PEG-ATO analogs on basal ECAR (intact cells) in human pancreatic cancer (MiaPaCa-2) cells.** Mito-PEG-ATO analogs increase intact cell ECAR while significantly decreasing basal OCR after 24 h treatment.


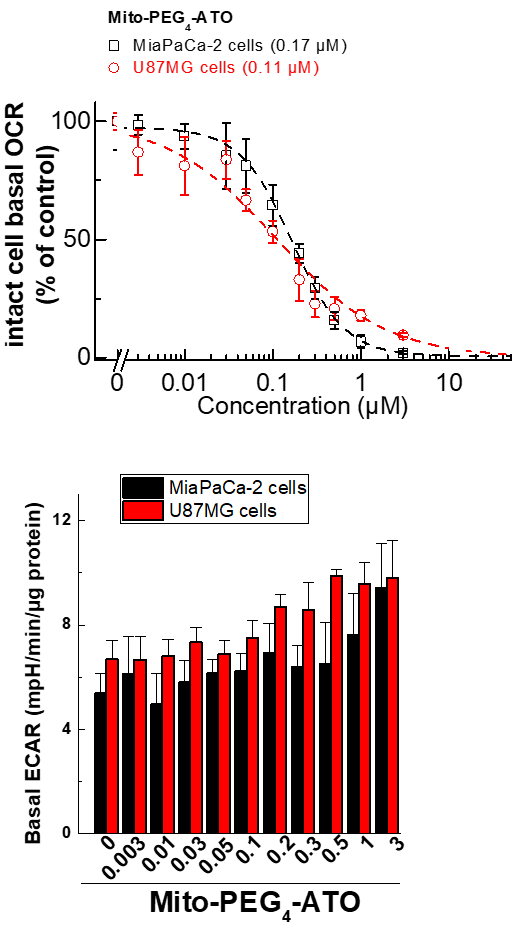


**Figure S10. Effects of Mito-PEG_4_-ATO on basal mitochondria OCR in human pancreatic cancer (MiaPaCa-2) cells and human brain cancer (U87MG) cells.**

**
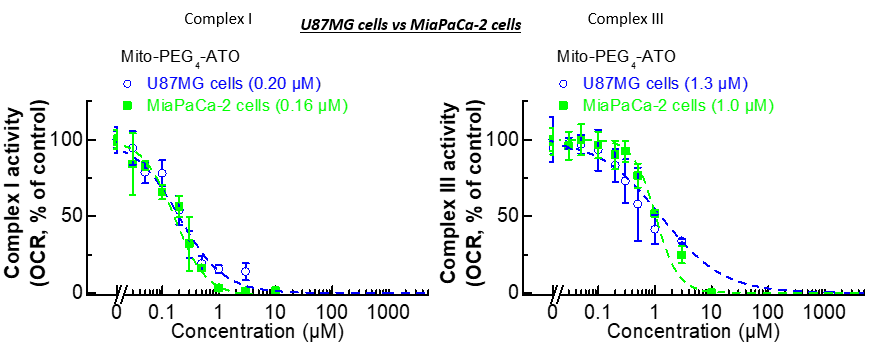
**

**Figure S11. Effects of Mito-PEG_4_-ATO on mitochondrial complex I and complex III activities in human pancreatic cancer (MiaPaCa-2) cells and human brain cancer (U87MG) cells.**

**
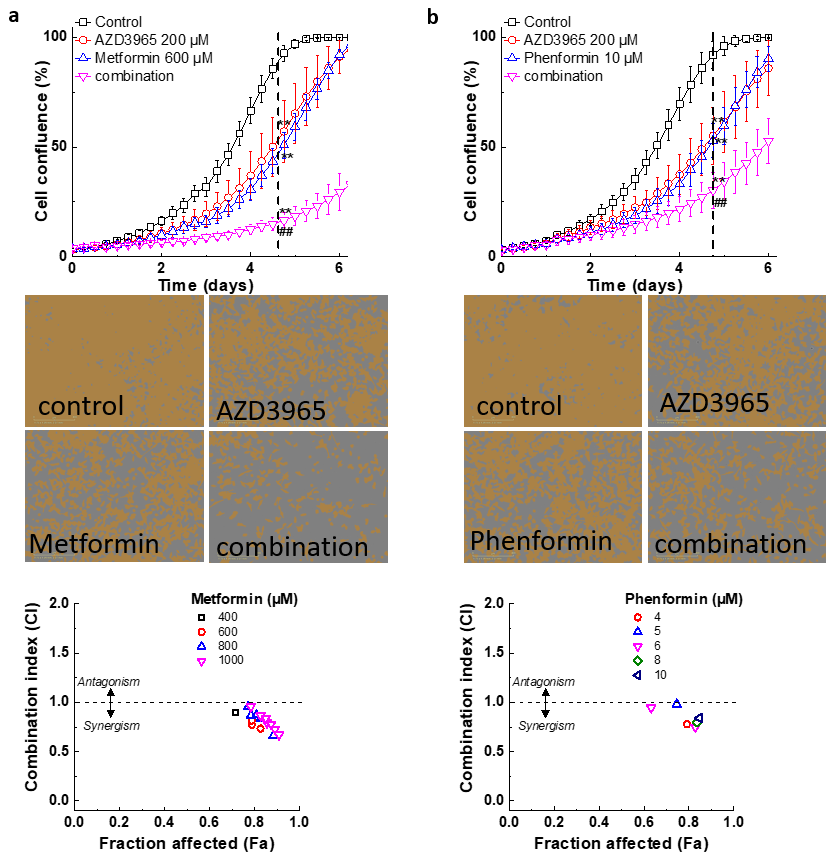
**

**Figure S12. Synergistic inhibition of cell proliferation by AZD-3965 and metformin or phenformin in human pancreatic cancer (MiaPaCa-2) cells. (Top)** MiaPaCa-2 cells were treated with AZD-3965 and metformin **(a)** or phenformin **(b)** independently and together at the indicated concentrations, and cell growth was monitored continuously. Data shown are the mean±SD (n=5). **(Middle)** Representative cell images are shown as a segmentation mask illustrated in brown when control cells reached ~90% confluence (vertical dashed line). ** *p*<0.01 vs control. ^##^*p*<0.01 vs AZD-3965 alone. **(Bottom)** Cell confluence (control cells reached ~90% confluence) is plotted against concentration for the synergistic calculation. Bottom panels (a, metformin; b, phenformin) show the combination index–fraction affected plots. The fraction affected parameter is used as a measure of the efficiency of the drug(s), with a value of 1 indicating complete inhibition of cell confluence and a value of 0 indicating the lack of effect on cell confluence. Data shown are the mean±SD.

**
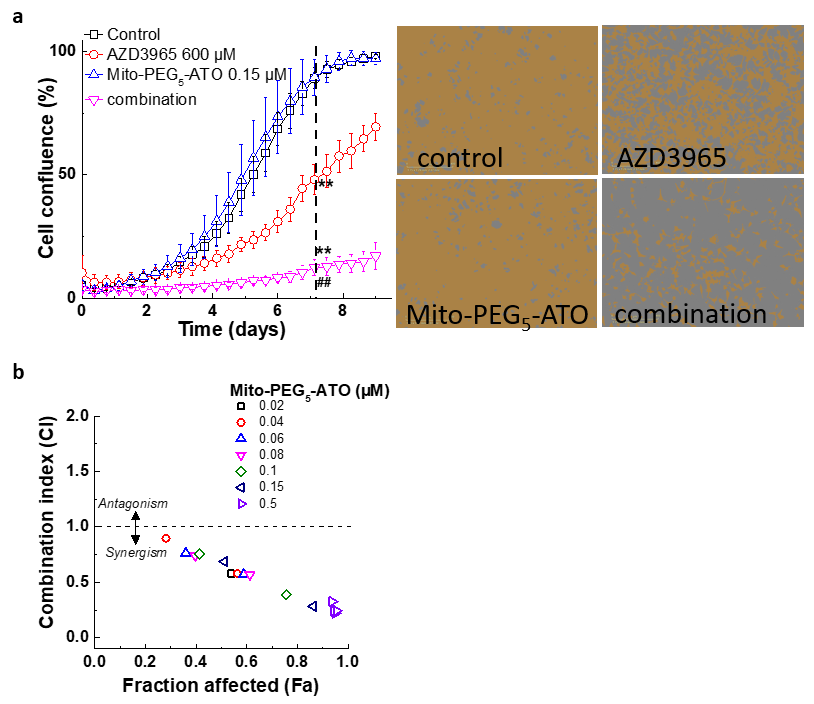
**

**Figure S13. Synergistic inhibition of cell proliferation by Mito-PEG_5_-ATO and AZD-3965 in human brain cancer (U87MG) cells. (a)** U87MG cells were treated with Mito-PEG_5_-ATO (0.15 µM) or AZD-3965 (600 µM) independently and together, and cell growth was monitored continuously. Data shown are the mean ± SD (n=5). Representative cell images are shown as a segmentation mask illustrated in brown when control cells reached ~90% confluence (vertical dashed line). ** *p*<0.01 vs control. ^##^ *p*<0.01 vs Mito-PEG_5_-ATO or AZD-3965 alone. **(b)** Cell confluence (control cells reached ~90% confluence) is plotted against concentration for the synergistic calculation. Panel b shows the combination index–fraction affected plots. Fraction affected parameter is used as a measure of the efficiency of the drug(s), with a value of 0 indicating complete inhibition of cell confluence and a value of 1 indicating the lack of effect on cell confluence. Data shown are the means ± SD.

**
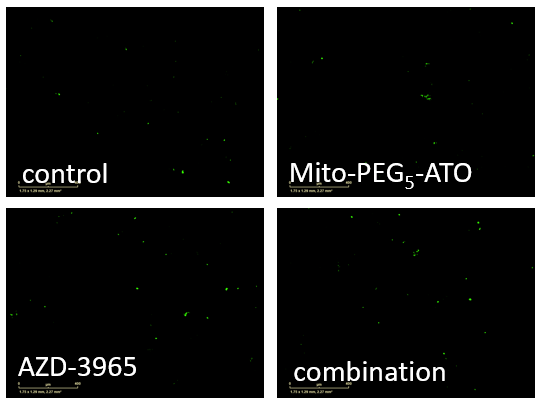
**

**Figure S14. Effects of Mito-PEG_5_-ATO and AZD-3965 on the extent of cell death in human pancreatic cancer (MiaPaCa-2) cells**. MiaPaCa-2 cells were treated with Mito-PEG_5_-ATO (0.04 µM) or AZD-3965 (200 µM) independently and together at the same concentrations used in Fig. 5 for 72 h, and cell death was monitored in real time with an IncuCyte Live-Cell Analysis System by SYTOX Green staining. The corresponding representative fluorescence images are shown SYTOX Green positive images after 72 h of treatment, as indicated.


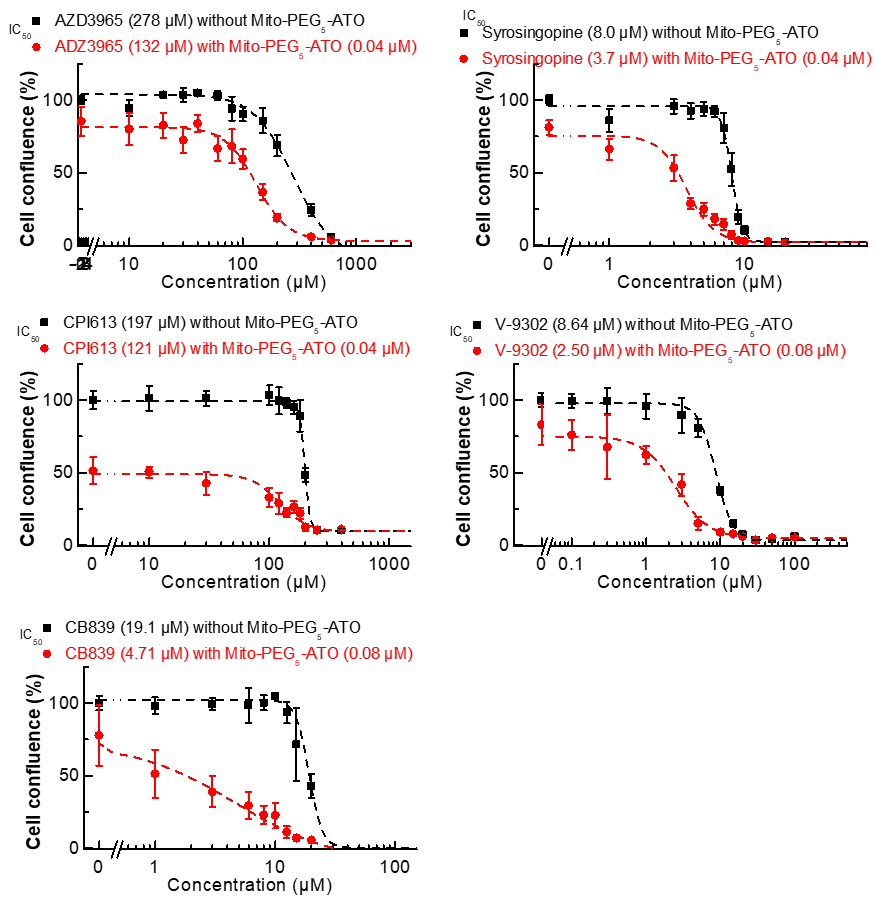


**Figure S15. A compounds screen identifies Mito-PEG_5_-ATO sensitizing human pancreatic cancer (MiaPaCa-2) cells to CPI-613, AZD-3965, syrosingopine, V-9032, and CB-839 treatment.** MiaPaCa-2 cells were treated with CPI-613, AZD-3965, syrosingopine, V-9032, or CB-839 independently or together with Mito-PEG_5_-ATO (as indicated), and cell growth was monitored continuously. The IC_50_ values with or without Mito-PEG_5_-ATO were determined at the point at which control cells reached ~90% confluence. These IC_50_ values were used to calculate percentage changes with or without Mito-PEG_5_-ATO and to generate the heat map shown in Figure 7d.


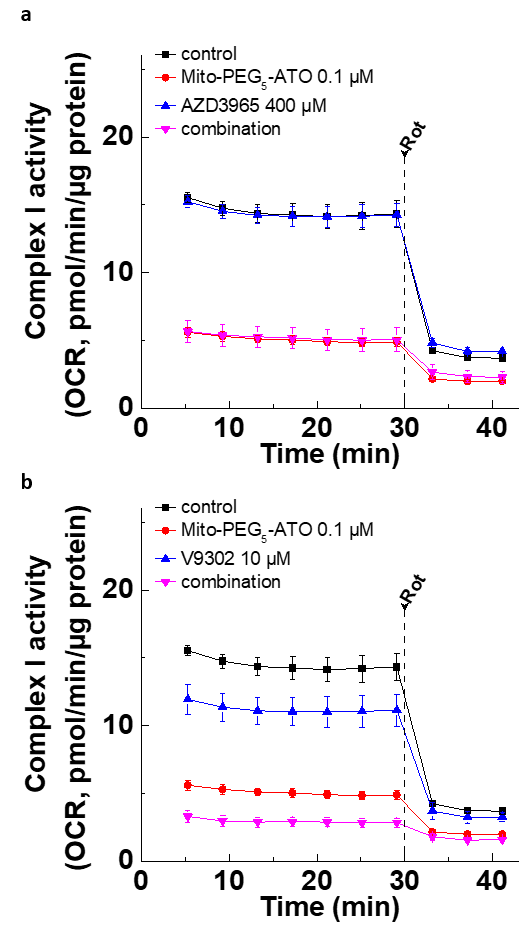


**Figure S16.** **The effect of combining Mito-PEG_5_-ATO with AZD-3965 or V-9302 on complex I- and complex III-induced oxygen consumption.** The effects of combining Mito-PEG_5_-ATO with AZD-3965 (a) or V-9302 (b) on complex I-dependent oxygen consumption were measured in MiaPaCa-2 cells after the cells were treated with Mito-PEG-ATO analogs as indicated for 24 h. Mitochondrial complex I activity was monitored by a Seahorse XF-96 Extracellular Flux Analyzer. Rotenone (complex I inhibitor) was acutely added and OCR assayed immediately. The mitochondrial complex I-dependent oxygen consumption represents rotenone-inhibitable OCR. Data shown are the mean±SD.


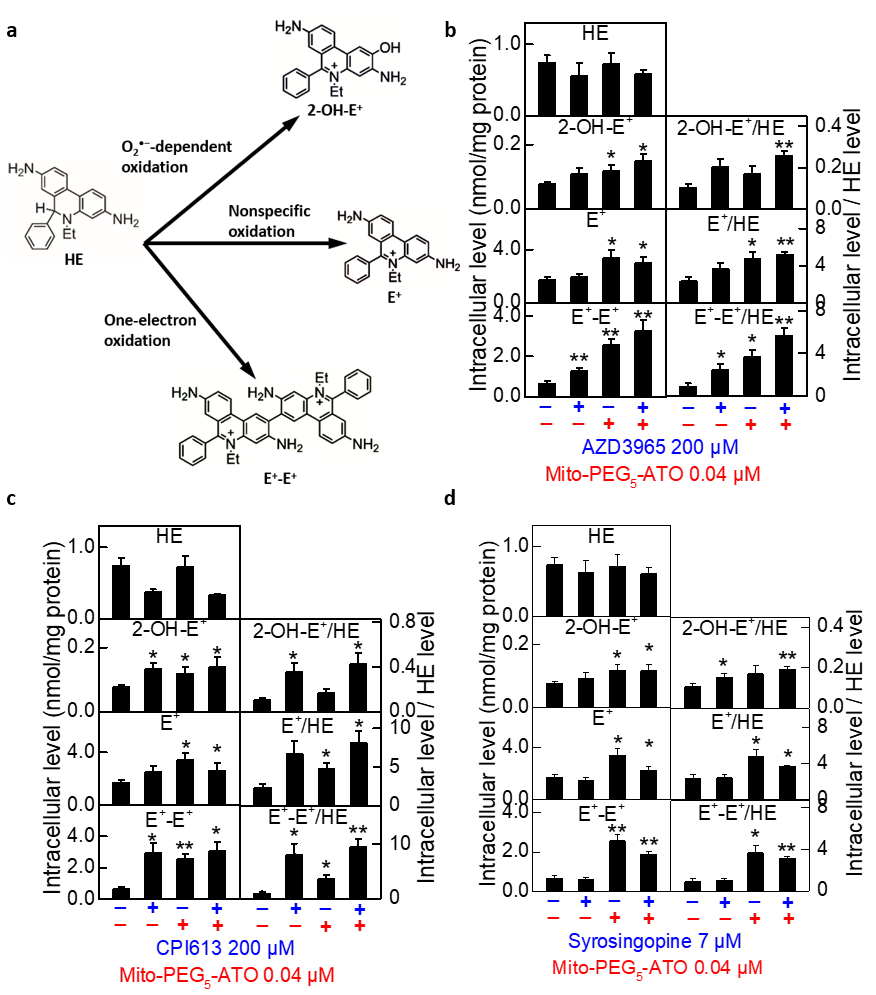


**Figure S17. Chemical structures of hydroethidine (HE), 2-hydroxyethidium (2-OH-E+), ethidium (E+), and diethidium (E+-E+) (a). Effects of Mito-PEG_5_-ATO and AZD-3965 (b), CPI-613 (c), or syrosingopine (d) on the hydroxylation and oxidation products of HE.** MiaPaCa-2 cells were treated with Mito-PEG_5_-ATO (0.05 µM) and AZD-3965 (200 µM) or CPI-613 (200 µM) for 24 h, followed by incubation with HE (10 µM) for 1 h. 2-OH-E^+^, E^+^, and E^+^-E^+^ were measured. Data represented as mean±SD, n=3. **p*<0.05, ***p*<0.01 vs control.

**
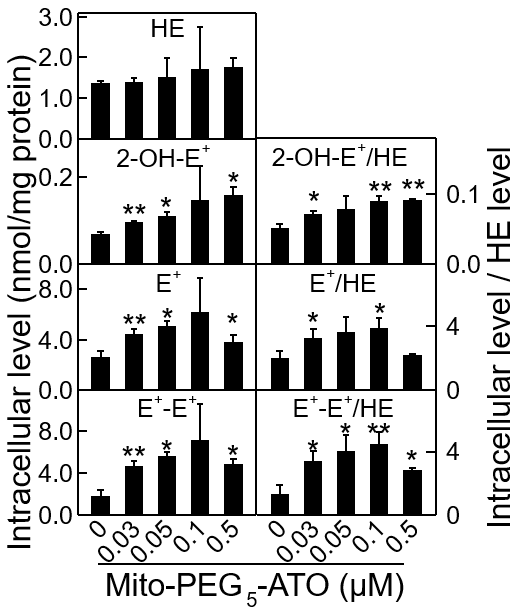
**

**Figure S18. Effect of Mito-PEG_5_-ATO on hydroxylation and oxidation products of hydroethidine (HE).** MiaPaCa-2 cells were pretreated with Mito-PEG_5_-ATO for 24 h, followed by incubation with HE (10 µM) for 1 h. 2-Hydroxyethidium (2-OH-E^+^) is a superoxide-specific product, ethidium (E^+^) is a nonspecific oxidation product, and diethidium (E^+^-E^+^) is a product of one-electron oxidation of the probe. Data represented as mean±SD, n=3. **p*<0.05, ***p*<0.01 vs control.

| **Table S1. Effects of Mito-PEG-ATO analogs on proliferation of human pancreatic cancer (MiaPaCa-2) cells.** The effects of Mito-PEG-ATO analogs on the proliferation of MiaPaCa-2 cells were monitored in the IncuCyte Live-Cell Analysis System. The IncuCyte analyzer provides real-time updates on cell confluence, based on segmentation of high definition-phase contrast images. Data are represented as a percentage of cell confluence when control cells reached ~90% confluence (vertical solid black line from Fig. 2). Data shown are the mean±SD, n=4 per treatment group. **p*< 0.05, ***p*< 0.01 vs control. | | | | | | | | | | | | | |
| --- | --- | --- | --- | --- | --- | --- | --- | --- | --- | --- | --- | --- | --- |
| **PEG_4_-ATO (µM)** | **Cell confluence**  **(% of control)** | | |  | **Mito-PEG_2_-ATO (µM)** | **Cell confluence**  **(% of control)** | | |  | **Mito-PEG_4_-ATO (µM)** | **Cell confluence**  **(% of control)** | | |
| 0 | 100.00 | ± | 2.42 |  | 0 | 100.00 | ± | 2.07 |  | 0 | 100.00 | ± | 2.52 |
| 1 | 96.77 | ± | 1.38 |  | 0.003 | 95.33 | ± | 8.02 |  | 0.005 | 100.53 | ± | 0.84 |
| 3 | 98.45 | ± | 3.90 |  | 0.01 | 101.09 | ± | 1.28 |  | 0.02 | 101.08 | ± | 1.00 |
| 5 | 92.22 | ± | 5.69* |  | 0.03 | 98.91 | ± | 2.86 |  | 0.05 | 100.72 | ± | 0.56 |
| 10 | 88.90 | ± | 4.72** |  | 0.05 | 101.49 | ± | 2.58 |  | 0.08 | 98.94 | ± | 1.26 |
| 15 | 79.02 | ± | 1.50** |  | 0.1 | 100.20 | ± | 2.82 |  | 0.16 | 29.68 | ± | 2.00** |
| 20 | 50.78 | ± | 3.81** |  | 0.2 | 91.59 | ± | 3.86** |  | 0.32 | 3.32 | ± | 0.41** |
| 30 | 33.44 | ± | 5.30** |  | 0.3 | 54.02 | ± | 2.10** |  | 0.48 | 3.11 | ± | 0.18** |
| 35 | 24.27 | ± | 3.59** |  | 0.5 | 5.85 | ± | 0.11** |  | 0.8 | 3.18 | ± | 0.25** |
| 40 | 17.96 | ± | 2.56** |  | 1 | 3.35 | ± | 0.09** |  | 1.6 | 2.81 | ± | 0.33** |
| 50 | 10.27 | ± | 1.93** |  | 3 | 3.28 | ± | 0.23** |  | 4.8 | 2.52 | ± | 0.24** |
|  |  |  |  |  | 10 | 2.85 | ± | 0.18** |  |  |  |  |  |
|  |  |  |  |  |  |  |  |  |  |  |  |  |  |
| **Mito-PEG_5_-ATO (µM)** | **Cell confluence**  **(% of control)** | | |  | **Mito-PEG_9_-ATO (µM)** | **Cell confluence**  **(% of control)** | | |  |  |  | | |
| 0 | 100.00 | ± | 2.07 |  | 0 | 100.00 | ± | 3.11 |  |  |  |  |  |
| 0.003 | 94.98 | ± | 6.42 |  | 0.01 | 99.63 | ± | 2.92 |  |  |  |  |  |
| 0.01 | 93.81 | ± | 4.28* |  | 0.03 | 99.03 | ± | 2.43 |  |  |  |  |  |
| 0.03 | 74.87 | ± | 4.01** |  | 0.1 | 81.02 | ± | 6.62** |  |  |  |  |  |
| 0.05 | 41.88 | ± | 3.37** |  | 0.15 | 60.68 | ± | 2.28** |  |  |  |  |  |
| 0.1 | 7.64 | ± | 1.01** |  | 0.2 | 25.97 | ± | 2.92** |  |  |  |  |  |
| 0.2 | 4.52 | ± | 0.12** |  | 0.25 | 14.32 | ± | 1.13** |  |  |  |  |  |
| 0.3 | 4.07 | ± | 0.44** |  | 0.3 | 10.81 | ± | 0.82** |  |  |  |  |  |
| 0.5 | 3.75 | ± | 0.08** |  | 0.4 | 7.81 | ± | 0.31** |  |  |  |  |  |
| 1 | 3.53 | ± | 0.24** |  | 0.5 | 5.89 | ± | 0.28** |  |  |  |  |  |
| 3 | 2.64 | ± | 0.07** |  | 1 | 4.84 | ± | 0.43** |  |  |  |  |  |
| 10 | 2.40 | ± | 0.14** |  | 5 | 3.30 | ± | 0.96** |  |  |  |  |  |

| **Table S2. Effects of Mito-PEG-ATO analogs on intact cell mitochondria oxygen consumption of human pancreatic cancer (MiaPaCa-2) cells.** MiaPaCa-2 cells were treated with Mito-PEG-ATO analogs for 24 h and concentration-dependent inhibition of mitochondrial respiration (OCR) in intact MiaPaCa-2 cells by Mito-PEG-ATO analogs were measured. Data are represented the average of the last three stable baseline OCR reading (Fig. 3a) used to calculate percentage of control and determine IC_50_ values for Fig. 3b. Data shown are the mean±SD, n=4 per treatment group. **p*<0.05, ***p*<0.01 vs control. | | | | | | | | | | | | | | | | | | | | | | | | | | | | | | | | | | | | | | | | | | | | | | | | | | | | | | | | | | | | | | | | | | | | | | | | | | | | | | | | | | | | | | | | | | | | | |  |  |
| --- | --- | --- | --- | --- | --- | --- | --- | --- | --- | --- | --- | --- | --- | --- | --- | --- | --- | --- | --- | --- | --- | --- | --- | --- | --- | --- | --- | --- | --- | --- | --- | --- | --- | --- | --- | --- | --- | --- | --- | --- | --- | --- | --- | --- | --- | --- | --- | --- | --- | --- | --- | --- | --- | --- | --- | --- | --- | --- | --- | --- | --- | --- | --- | --- | --- | --- | --- | --- | --- | --- | --- | --- | --- | --- | --- | --- | --- | --- | --- | --- | --- | --- | --- | --- | --- | --- | --- | --- | --- | --- | --- | --- | --- | --- | --- |
| **ATO (µM)** | | | | | | | | **OCR (pmol/min/µg protein)** | | | | | | | | | | | | | | | |  | | **PEG_4_-ATO (µM)** | | | | | | | | | | | | | | | **OCR**  **(pmol/min/µg protein)** | | | | | | | | | | | | | | | | | |  | **Mito-PEG_2_-ATO (µM)** | | | | | | | | | | | | | | | | | **OCR**  **(pmol/min/µg protein)** | | | | | | | | | | | | | | | | |  |  |
| 0 | | | | | | | | 13.31 | | | | | | | | | ± | | 0.63 | | | | |  | | 0 | | | | | | | | | | | | | | | 13.04 | | | | | | | ± | | | | | 0.96 | | | | | |  | 0 | | | | | | | | | | | | | | | | | 17.59 | | | | | | | | ± | | 0.78 | | | | | | |  |  |
| 1 | | | | | | | | 11.69 | | | | | | | | | ± | | 0.56* | | | | |  | | 1 | | | | | | | | | | | | | | | 14.02 | | | | | | | ± | | | | | 0.46 | | | | | |  | 0.01 | | | | | | | | | | | | | | | | | 16.21 | | | | | | | | ± | | 0.54* | | | | | | |  |  |
| 3 | | | | | | | | 11.27 | | | | | | | | | ± | | 0.33** | | | | |  | | 3 | | | | | | | | | | | | | | | 11.47 | | | | | | | ± | | | | | 1.61 | | | | | |  | 0.1 | | | | | | | | | | | | | | | | | 14.62 | | | | | | | | ± | | 1.40** | | | | | | |  |  |
| 10 | | | | | | | | 4.45 | | | | | | | | | ± | | 0.45** | | | | |  | | 10 | | | | | | | | | | | | | | | 8.99 | | | | | | | ± | | | | | 0.68** | | | | | |  | 0.2 | | | | | | | | | | | | | | | | | 14.19 | | | | | | | | ± | | 0.55** | | | | | | |  |  |
| 15 | | | | | | | | 3.40 | | | | | | | | | ± | | 1.10** | | | | |  | | 15 | | | | | | | | | | | | | | | 8.66 | | | | | | | ± | | | | | 0.09** | | | | | |  | 0.3 | | | | | | | | | | | | | | | | | 13.58 | | | | | | | | ± | | 0.62** | | | | | | |  |  |
| 17.5 | | | | | | | | 2.08 | | | | | | | | | ± | | 0.21** | | | | |  | | 17.5 | | | | | | | | | | | | | | | 8.14 | | | | | | | ± | | | | | 0.52** | | | | | |  | 0.4 | | | | | | | | | | | | | | | | | 12.73 | | | | | | | | ± | | 1.07** | | | | | | |  |  |
| 20 | | | | | | | | 1.80 | | | | | | | | | ± | | 0.30** | | | | |  | | 20 | | | | | | | | | | | | | | | 6.77 | | | | | | | ± | | | | | 0.62** | | | | | |  | 0.5 | | | | | | | | | | | | | | | | | 10.74 | | | | | | | | ± | | 1.21** | | | | | | |  |  |
| 22.5 | | | | | | | | 2.05 | | | | | | | | | ± | | 0.28** | | | | |  | | 22.5 | | | | | | | | | | | | | | | 6.79 | | | | | | | ± | | | | | 0.25** | | | | | |  | 0.75 | | | | | | | | | | | | | | | | | 8.54 | | | | | | | | ± | | 0.54** | | | | | | |  |  |
| 25 | | | | | | | | 2.46 | | | | | | | | | ± | | 0.22** | | | | |  | | 25 | | | | | | | | | | | | | | | 5.93 | | | | | | | ± | | | | | 0.46** | | | | | |  | 1 | | | | | | | | | | | | | | | | | 6.82 | | | | | | | | ± | | 1.12** | | | | | | |  |  |
| 30 | | | | | | | | 1.99 | | | | | | | | | ± | | 0.20** | | | | |  | | 30 | | | | | | | | | | | | | | | 6.26 | | | | | | | ± | | | | | 0.39** | | | | | |  | 3 | | | | | | | | | | | | | | | | | 1.26 | | | | | | | | ± | | 0.36** | | | | | | |  |  |
| 40 | | | | | | | | 2.11 | | | | | | | | | ± | | 0.45** | | | | |  | | 40 | | | | | | | | | | | | | | | 4.53 | | | | | | | ± | | | | | 0.73** | | | | | |  | 10 | | | | | | | | | | | | | | | | | -0.10 | | | | | | | | ± | | 0.52** | | | | | | |  |  |
| 50 | | | | | | | | 0.93 | | | | | | | | | ± | | 1.71** | | | | |  | | 50 | | | | | | | | | | | | | | | 2.22 | | | | | | | ± | | | | | 0.22** | | | | | |  |  | | | | | | | | | | | | | | | | |  | | | | | | | |  | |  | | | | | | |  |  |
|  | | | | | | | |  | | | | | | | | |  | |  | | | | |  | |  | | | | | | | | | | | | | | |  | | | | | | |  | | | | |  | | | | | |  |  | | | | | | | | | | | | | | | | |  | | | | | | | |  | |  | | | | | | |  |  |
| **Mito-PEG_4_-ATO (µM)** | | | | | | | | **OCR**  **(pmol/min/µg protein)** | | | | | | | | | | | | | | | |  | | **Mito-PEG_5_-ATO (µM)** | | | | | | | | | | | | | | | **OCR**  **(pmol/min/µg protein)** | | | | | | | | | | | | | | | | | |  | **Mito-PEG_9_-ATO (µM)** | | | | | | | | | | | | | | | | | **OCR**  **(pmol/min/µg protein)** | | | | | | | | | | | | | | | | |  |  |
| 0 | | | | | | | | 20.74 | | | | | | | | | ± | | 2.50 | | | | |  | | 0 | | | | | | | | | | | | | | | 15.06 | | | | | | | ± | | | | | 1.42 | | | | | |  | 0 | | | | | | | | | | | | | | | | | 15.03 | | | | | | | | ± | | 1.32 | | | | | | |  |  |
| 0.003 | | | | | | | | 20.44 | | | | | | | | | ± | | 0.88 | | | | |  | | 0.003 | | | | | | | | | | | | | | | 13.36 | | | | | | | ± | | | | | 0.82 | | | | | |  | 0.01 | | | | | | | | | | | | | | | | | 13.41 | | | | | | | | ± | | 1.12 | | | | | | |  |  |
| 0.01 | | | | | | | | 17.79 | | | | | | | | | ± | | 3.36 | | | | |  | | 0.01 | | | | | | | | | | | | | | | 12.51 | | | | | | | ± | | | | | 1.56 | | | | | |  | 0.03 | | | | | | | | | | | | | | | | | 12.24 | | | | | | | | ± | | 1.37* | | | | | | |  |  |
| 0.03 | | | | | | | | 17.72 | | | | | | | | | ± | | 2.89 | | | | |  | | 0.03 | | | | | | | | | | | | | | | 9.91 | | | | | | | ± | | | | | 1.26** | | | | | |  | 0.1 | | | | | | | | | | | | | | | | | 7.60 | | | | | | | | ± | | 0.36** | | | | | | |  |  |
| 0.05 | | | | | | | | 16.82 | | | | | | | | | ± | | 2.38 | | | | |  | | 0.05 | | | | | | | | | | | | | | | 9.30 | | | | | | | ± | | | | | 1.55** | | | | | |  | 0.2 | | | | | | | | | | | | | | | | | 3.64 | | | | | | | | ± | | 0.16** | | | | | | |  |  |
| 0.1 | | | | | | | | 13.38 | | | | | | | | | ± | | 1.80** | | | | |  | | 0.075 | | | | | | | | | | | | | | | 8.05 | | | | | | | ± | | | | | 1.16** | | | | | |  | 0.3 | | | | | | | | | | | | | | | | | 1.87 | | | | | | | | ± | | 0.11** | | | | | | |  |  |
| 0.2 | | | | | | | | 9.15 | | | | | | | | | ± | | 0.86** | | | | |  | | 0.1 | | | | | | | | | | | | | | | 5.43 | | | | | | | ± | | | | | 0.30** | | | | | |  | 0.4 | | | | | | | | | | | | | | | | | 1.43 | | | | | | | | ± | | 0.26** | | | | | | |  |  |
| 0.3 | | | | | | | | 6.10 | | | | | | | | | ± | | 0.97** | | | | |  | | 0.3 | | | | | | | | | | | | | | | 0.36 | | | | | | | ± | | | | | 2.70** | | | | | |  | 0.5 | | | | | | | | | | | | | | | | | 1.22 | | | | | | | | ± | | 0.19** | | | | | | |  |  |
| 0.5 | | | | | | | | 3.29 | | | | | | | | | ± | | 0.77** | | | | |  | | 0.5 | | | | | | | | | | | | | | | 1.83 | | | | | | | ± | | | | | 0.45** | | | | | |  | 1 | | | | | | | | | | | | | | | | | 1.18 | | | | | | | | ± | | 0.32** | | | | | | |  |  |
| 1 | | | | | | | | 1.41 | | | | | | | | | ± | | 0.55** | | | | |  | | 1 | | | | | | | | | | | | | | | 1.40 | | | | | | | ± | | | | | 0.18** | | | | | |  | 3 | | | | | | | | | | | | | | | | | 0.96 | | | | | | | | ± | | 0.14** | | | | | | |  |  |
| 3 | | | | | | | | 0.43 | | | | | | | | | ± | | 0.15** | | | | |  | | 3 | | | | | | | | | | | | | | | 0.84 | | | | | | | ± | | | | | 0.13** | | | | | |  | 5 | | | | | | | | | | | | | | | | | 0.01 | | | | | | | | ± | | 0.24** | | | | | | |  |  |
| 10 | | | | | | | | 0.42 | | | | | | | | | ± | | 0.12** | | | | |  | | 10 | | | | | | | | | | | | | | | 0.55 | | | | | | | ± | | | | | 0.51** | | | | | |  |  | | | | | | | | | | | | | | | | |  | | | | | | | |  | |  | | | | | | |  |  |
| **Table S3.** **Effects of Mito-PEG-ATO analogs on oxygen consumption by mitochondrial complex I in human pancreatic cancer (MiaPaCa-2) cells; related to Figure 4a.** Effects of Mito-PEG-ATO analogs on complex I- and complex III-dependent oxygen consumption were measured in MiaPaCa-2 cells. Data represent complex I and complex III activity (% of control, calculated from Fig. 4b) used to determine the IC_50_ values in Fig. 4b. Data shown are the mean±SD, n=4 per treatment group. **p*<0.05, ***p*<0.01 vs control. | | | | | | | | | | | | | | | | | | | | | | | | | | | | | | | | | | | | | | | | | | | | | | | | | | | | | | | | | | | | | | | | | | | | | | | | | | | | | | | | | | | | | | | | | | | | | | |  |
| **Complex I activity** | | | | | | | | | | | | | | | | | | | | | | | | | | | | | | | | | | | | | | | | | | | | | | | | | | | | | | | | | | | | | | | | | | | | | | | | | | | | | | | | | | | | | | | | | | | | | | |  |
| **Mito-PEG_2_-ATO (µM)** | | | | **(OCR, % of control)** | | | | | | | | | | | |  | | **Mito-PEG_4_-ATO (µM)** | | | | | | | | | | | | **(OCR, % of control)** | | | | | | | | | | | |  | | **Mito-PEG_5_-ATO (µM)** | | | | | | | | | | | | | | **(OCR, % of control)** | | | | | | | | | | | | |  | **Mito-PEG_9_-ATO (µM)** | | | | | | | | | | | | **(OCR, % of control)** | | | | | | | | | | |  |
| 0 | | | | 100.0 | | | | | ± | | 8.3 | | | | |  | | 0 | | | | | | | | | | | | 100.0 | | | | | ± | | 6.4 | | | | |  | | 0 | | | | | | | | | | | | | | 100.0 | | | | | | | ± | 8.3 | | | | |  | 0 | | | | | | | | | | | | 100.0 | | | | | ± | | | 11.3 | | |  |
| 0.01 | | | | 89.5 | | | | | ± | | 6.0 | | | | |  | | 0.02 | | | | | | | | | | | | 98.6 | | | | | ± | | 4.4 | | | | |  | | 0.003 | | | | | | | | | | | | | | 95.2 | | | | | | | ± | 5.1 | | | | |  | 0.003 | | | | | | | | | | | | 98.2 | | | | | ± | | | 5.4 | | |  |
| 0.1 | | | | 73.5 | | | | | ± | | 14.1* | | | | |  | | 0.05 | | | | | | | | | | | | 84.0 | | | | | ± | | 20.1 | | | | |  | | 0.01 | | | | | | | | | | | | | | 96.2 | | | | | | | ± | 6.4 | | | | |  | 0.01 | | | | | | | | | | | | 91.2 | | | | | ± | | | 9.0 | | |  |
| 0.2 | | | | 78.5 | | | | | ± | | 3.5** | | | | |  | | 0.08 | | | | | | | | | | | | 83.0 | | | | | ± | | 2.8** | | | | |  | | 0.03 | | | | | | | | | | | | | | 62.1 | | | | | | | ± | 4.0** | | | | |  | 0.03 | | | | | | | | | | | | 76.0 | | | | | ± | | | 4.2** | | |  |
| 0.3 | | | | 57.9 | | | | | ± | | 10** | | | | |  | | 0.16 | | | | | | | | | | | | 65.7 | | | | | ± | | 4.7** | | | | |  | | 0.05 | | | | | | | | | | | | | | 63.3 | | | | | | | ± | 3.2** | | | | |  | 0.05 | | | | | | | | | | | | 66.5 | | | | | ± | | | 12** | | |  |
| 0.4 | | | | 59.1 | | | | | ± | | 7.4** | | | | |  | | 0.32 | | | | | | | | | | | | 56.2 | | | | | ± | | 6.7** | | | | |  | | 0.075 | | | | | | | | | | | | | | 50.4 | | | | | | | ± | 5.6** | | | | |  | 0.1 | | | | | | | | | | | | 51.9 | | | | | ± | | | 9.4** | | |  |
| 0.5 | | | | 47.4 | | | | | ± | | 4.4** | | | | |  | | 0.5 | | | | | | | | | | | | 32.0 | | | | | ± | | 18** | | | | |  | | 0.1 | | | | | | | | | | | | | | 42.6 | | | | | | | ± | 6.2** | | | | |  | 0.2 | | | | | | | | | | | | 22.1 | | | | | ± | | | 2.0** | | |  |
| 0.75 | | | | 28.5 | | | | | ± | | 5.4** | | | | |  | | 0.8 | | | | | | | | | | | | 16.0 | | | | | ± | | 1.6** | | | | |  | | 0.3 | | | | | | | | | | | | | | 9.2 | | | | | | | ± | 1.9** | | | | |  | 0.3 | | | | | | | | | | | | 8.0 | | | | | ± | | | 0.8** | | |  |
| 1 | | | | 14.6 | | | | | ± | | 3.2** | | | | |  | | 1.6 | | | | | | | | | | | | 3.3 | | | | | ± | | 0.6** | | | | |  | | 0.5 | | | | | | | | | | | | | | 5.2 | | | | | | | ± | 5.0** | | | | |  | 0.4 | | | | | | | | | | | | 3.6 | | | | | ± | | | 1.3** | | |  |
| 3 | | | | 1.3 | | | | | ± | | 1.3** | | | | |  | | 5 | | | | | | | | | | | | 1.1 | | | | | ± | | 1.8** | | | | |  | | 1 | | | | | | | | | | | | | | 1.0 | | | | | | | ± | 1.7** | | | | |  | 0.5 | | | | | | | | | | | | 1.5 | | | | | ± | | | 1.1** | | |  |
|  | | | |  | | | | |  | |  | | | | |  | |  | | | | | | | | | | | |  | | | | |  | |  | | | | |  | | 3 | | | | | | | | | | | | | | 0.4 | | | | | | | ± | 4.5** | | | | |  | 0.75 | | | | | | | | | | | | 1.7 | | | | | ± | | | 0.1** | | |  |
|  | | | |  | | | | |  | |  | | | | |  | |  | | | | | | | | | | | |  | | | | |  | |  | | | | |  | |  | | | | | | | | | | | | | |  | | | | | | |  |  | | | | |  | 1 | | | | | | | | | | | | 1.3 | | | | | ± | | | 0.6** | | |  |
|  | | | |  | | | | |  | |  | | | | |  | |  | | | | | | | | | | | |  | | | | |  | |  | | | | |  | |  | | | | | | | | | | | | | |  | | | | | | |  |  | | | | |  |  | | | | | | | | | | | |  | | | | |  | | |  | | |  |
| **Complex III activity** | | | | | | | | | | | | | | | | | | | | | | | | | | | | | | | | | | | | | | | | | | | | | | | | | | | | | | | | | | | | | | | | | | | | | | | | | | | | | | | | | | | | | | | | | | | | | | |  |
| **Mito-PEG_2_-ATO (µM)** | | | | **(OCR, % of control)** | | | | | | | | | | | |  | | **Mito-PEG_4_-ATO (µM)** | | | | | | | | | | | | **(OCR, % of control)** | | | | | | | | | | | |  | | **Mito-PEG_5_-ATO (µM)** | | | | | | | | | | | | | | **(OCR, % of control)** | | | | | | | | | | | | |  | **Mito-PEG_9_-ATO (µM)** | | | | | | | | | | | | **(OCR, % of control)** | | | | | | | | | | |  |
| 0 | | | | 100.0 | | | | | ± | | 13.0 | | | | |  | | 0 | | | | | | | | | | | | 100.0 | | | | | ± | | 7.6 | | | | |  | | 0 | | | | | | | | | | | | | | 100.0 | | | | | | | ± | 13.0 | | | | |  | 0 | | | | | | | | | | | | 100.0 | | | | | ± | | | 8.5 | | |  |
| 0.01 | | | | 103.6 | | | | | ± | | 10.1 | | | | |  | | 0.02 | | | | | | | | | | | | 98.8 | | | | | ± | | 8.6 | | | | |  | | 0.003 | | | | | | | | | | | | | | 94.9 | | | | | | | ± | 3.1 | | | | |  | 0.003 | | | | | | | | | | | | 101.6 | | | | | ± | | | 2.9 | | |  |
| 0.1 | | | | 95.3 | | | | | ± | | 6.5 | | | | |  | | 0.05 | | | | | | | | | | | | 96.0 | | | | | ± | | 9.3 | | | | |  | | 0.01 | | | | | | | | | | | | | | 94.3 | | | | | | | ± | 13.8 | | | | |  | 0.01 | | | | | | | | | | | | 95.6 | | | | | ± | | | 6.4 | | |  |
| 0.2 | | | | 101.4 | | | | | ± | | 10.9 | | | | |  | | 0.08 | | | | | | | | | | | | 99.9 | | | | | ± | | 10.0 | | | | |  | | 0.03 | | | | | | | | | | | | | | 84.2 | | | | | | | ± | 7.7 | | | | |  | 0.03 | | | | | | | | | | | | 96.5 | | | | | ± | | | 1.2 | | |  |
| 0.3 | | | | 99.3 | | | | | ± | | 12.9 | | | | |  | | 0.16 | | | | | | | | | | | | 96.5 | | | | | ± | | 8.7 | | | | |  | | 0.05 | | | | | | | | | | | | | | 89.9 | | | | | | | ± | 9.6 | | | | |  | 0.05 | | | | | | | | | | | | 100.1 | | | | | ± | | | 4.8 | | |  |
| 0.4 | | | | 99.1 | | | | | ± | | 4.2 | | | | |  | | 0.32 | | | | | | | | | | | | 90.1 | | | | | ± | | 9.4 | | | | |  | | 0.075 | | | | | | | | | | | | | | 89.1 | | | | | | | ± | 10.4 | | | | |  | 0.1 | | | | | | | | | | | | 95.3 | | | | | ± | | | 5.9 | | |  |
| 0.5 | | | | 88.9 | | | | | ± | | 4.2 | | | | |  | | 0.5 | | | | | | | | | | | | 92.4 | | | | | ± | | 7.1 | | | | |  | | 0.1 | | | | | | | | | | | | | | 87.5 | | | | | | | ± | 16.8 | | | | |  | 0.2 | | | | | | | | | | | | 93.6 | | | | | ± | | | 13.6 | | |  |
| 0.75 | | | | 75.4 | | | | | ± | | 6.0* | | | | |  | | 0.8 | | | | | | | | | | | | 76.5 | | | | | ± | | 7.8** | | | | |  | | 0.3 | | | | | | | | | | | | | | 61.6 | | | | | | | ± | 14** | | | | |  | 0.3 | | | | | | | | | | | | 92.1 | | | | | ± | | | 6.2 | | |  |
| 1 | | | | 64.3 | | | | | ± | | 11** | | | | |  | | 1.6 | | | | | | | | | | | | 51.9 | | | | | ± | | 2.5** | | | | |  | | 0.5 | | | | | | | | | | | | | | 38.4 | | | | | | | ± | 10** | | | | |  | 0.4 | | | | | | | | | | | | 72.1 | | | | | ± | | | 5.5** | | |  |
| 3 | | | | 15.0 | | | | | ± | | 1.3** | | | | |  | | 5 | | | | | | | | | | | | 24.8 | | | | | ± | | 5.5** | | | | |  | | 1 | | | | | | | | | | | | | | 17.8 | | | | | | | ± | 5.0** | | | | |  | 0.5 | | | | | | | | | | | | 48.1 | | | | | ± | | | 4.8** | | |  |
|  | | | |  | | | | |  | |  | | | | |  | |  | | | | | | | | | | | |  | | | | |  | |  | | | | |  | | 3 | | | | | | | | | | | | | | 8.0 | | | | | | | ± | 4.7** | | | | |  | 0.75 | | | | | | | | | | | | 16.6 | | | | | ± | | | 4.0** | | |  |
|  | | | |  | | | | |  | |  | | | | |  | |  | | | | | | | | | | | |  | | | | |  | |  | | | | |  | |  | | | | | | | | | | | | | |  | | | | | | |  |  | | | | |  | 1 | | | | | | | | | | | | 4.7 | | | | | ± | | | 1.5** | | |  |
|  | | | | | | | | | | | | | | | | | | | | | | | | | | | | | | | | | | | | | | | | | | | | | | | | | | | | | | | | | | | | | | | | | | | | | | | | | | | | | | | | | | | | | | | | | | | | | | |  |
| **Table S4. Effect of Mito-PEG_5_-ATO in combination with syrosingopine and with AZD-3965 on intracellular ATP level (nmol ATP/mg protein), 24 h treatment (mean±SD, n=4)** | | | | | | | | | | | | | | | | | | | | | | | | | | | | | | | | | | | | | | | | | | | | | | | | | | | | | | | | | | | | | | | | | | | | | | | | | | | | | | | | | | | | | | | | | | | | | | |  |
|  | | **Mito-PEG_5_-ATO (µM)** | | | | | | | | | | | | | | | | | | | | | | | | | | | | | | | | | | | | | | | | | | | | | | | | | | | | | | | | | | | | | | | | | | | | | | | | | | | | | | | | | | | | | | | | | | | | |  |
| **Syrosingopine (µM)** | | **0** | | | | | | | | | | | |  | **0.02** | | | | | | | | | | | |  | | **0.04** | | | | | | | | |  | | **0.06** | | | | | | | | | |  | | **0.08** | | | | | | | | |  | | **0.1** | | | | | | | | | |  | | **0.15** | | | | | |  | **0.5** | | | | | | | | | | | | |  |
| 0 | | 29.43 | | | | ± | | | | 3.77 | | | |  | 33.08 | | | | | | ± | | 1.61 | | | |  | | 29.16 | | | ± | | 2.37 | | | |  | | 28.90 | | | | | ± | | 2.79 | | |  | | 27.83 | | | ± | 1.40 | | | | |  | | 27.64 | | | | ± | | 2.82 | | | |  | | 28.11 | | ± | | 2.97 | |  | 26.12 | | | | | | ± | | | 2.42 | | | |  |
| 1 | | 29.40 | | | | ± | | | | 2.57 | | | |  | 33.23 | | | | | | ± | | 1.49 | | | |  | | 29.37 | | | ± | | 1.67 | | | |  | | 28.12 | | | | | ± | | 1.74 | | |  | | 28.24 | | | ± | 1.84 | | | | |  | | 27.09 | | | | ± | | 2.46 | | | |  | | 28.32 | | ± | | 2.23 | |  | 25.90 | | | | | | ± | | | 1.81 | | | |  |
| 3 | | 30.31 | | | | ± | | | | 3.51 | | | |  | 33.21 | | | | | | ± | | 1.87 | | | |  | | 28.91 | | | ± | | 1.90 | | | |  | | 29.17 | | | | | ± | | 2.39 | | |  | | 28.92 | | | ± | 2.28 | | | | |  | | 27.75 | | | | ± | | 2.20 | | | |  | | 28.40 | | ± | | 3.17 | |  | 28.17 | | | | | | ± | | | 3.44 | | | |  |
| 4 | | 29.92 | | | | ± | | | | 3.46 | | | |  | 32.85 | | | | | | ± | | 0.90 | | | |  | | 28.58 | | | ± | | 2.27 | | | |  | | 28.49 | | | | | ± | | 2.04 | | |  | | 28.03 | | | ± | 1.65 | | | | |  | | 28.62 | | | | ± | | 1.68 | | | |  | | 29.45 | | ± | | 2.38 | |  | 27.89 | | | | | | ± | | | 1.99 | | | |  |
| 5 | | 30.42 | | | | ± | | | | 3.46 | | | |  | 33.62 | | | | | | ± | | 0.61 | | | |  | | 29.28 | | | ± | | 2.05 | | | |  | | 30.24 | | | | | ± | | 2.51 | | |  | | 29.46 | | | ± | 2.40 | | | | |  | | 29.08 | | | | ± | | 2.26 | | | |  | | 29.49 | | ± | | 1.71 | |  | 29.18 | | | | | | ± | | | 1.81 | | | |  |
| 6 | | 30.16 | | | | ± | | | | 2.52 | | | |  | 33.73 | | | | | | ± | | 1.22 | | | |  | | 29.64 | | | ± | | 1.24 | | | |  | | 30.51 | | | | | ± | | 1.53 | | |  | | 28.89 | | | ± | 2.87 | | | | |  | | 30.21 | | | | ± | | 1.55 | | | |  | | 29.86 | | ± | | 1.95 | |  | 30.85 | | | | | | ± | | | 1.33 | | | |  |
| 7 | | 31.07 | | | | ± | | | | 3.15 | | | |  | 32.07 | | | | | | ± | | 1.90 | | | |  | | 29.95 | | | ± | | 1.08 | | | |  | | 31.17 | | | | | ± | | 1.80 | | |  | | 31.65 | | | ± | 2.45 | | | | |  | | 32.30 | | | | ± | | 1.94 | | | |  | | 32.35 | | ± | | 2.53 | |  | 31.21 | | | | | | ± | | | 1.31 | | | |  |
| 8 | | 31.92 | | | | ± | | | | 2.74 | | | |  | 32.26 | | | | | | ± | | 1.05 | | | |  | | 30.48 | | | ± | | 1.48 | | | |  | | 32.03 | | | | | ± | | 2.06 | | |  | | 32.12 | | | ± | 2.16 | | | | |  | | 31.26 | | | | ± | | 0.93 | | | |  | | 31.72 | | ± | | 2.34 | |  | 31.78 | | | | | | ± | | | 2.36 | | | |  |
| 9 | | 32.59 | | | | ± | | | | 2.23 | | | |  | 31.75 | | | | | | ± | | 2.62 | | | |  | | 32.09 | | | ± | | 1.78 | | | |  | | 32.61 | | | | | ± | | 2.02 | | |  | | 32.18 | | | ± | 1.45 | | | | |  | | 32.76 | | | | ± | | 2.44 | | | |  | | 32.57 | | ± | | 2.27 | |  | 32.12 | | | | | | ± | | | 1.99 | | | |  |
| 10 | | 32.30 | | | | ± | | | | 3.28 | | | |  | 29.50 | | | | | | ± | | 1.75 | | | |  | | 30.41 | | | ± | | 1.31 | | | |  | | 31.75 | | | | | ± | | 1.94 | | |  | | 31.76 | | | ± | 1.31 | | | | |  | | 32.90 | | | | ± | | 1.08 | | | |  | | 32.11 | | ± | | 2.75 | |  | 31.69 | | | | | | ± | | | 2.47 | | | |  |
| 15 | | 33.63 | | | | ± | | | | 2.03 | | | |  | 26.42 | | | | | | ± | | 2.35 | | | |  | | 32.02 | | | ± | | 1.47 | | | |  | | 31.78 | | | | | ± | | 2.05 | | |  | | 30.67 | | | ± | 0.90 | | | | |  | | 30.01 | | | | ± | | 1.79 | | | |  | | 29.92 | | ± | | 1.33 | |  | 28.40 | | | | | | ± | | | 0.98 | | | |  |
| 20 | | 30.18 | | | | ± | | | | 3.38 | | | |  | 24.05 | | | | | | ± | | 4.61 | | | |  | | 28.65 | | | ± | | 4.07 | | | |  | | 29.49 | | | | | ± | | 2.87 | | |  | | 27.99 | | | ± | 3.16 | | | | |  | | 27.80 | | | | ± | | 2.64 | | | |  | | 26.45 | | ± | | 2.84 | |  | 25.00 | | | | | | ± | | | 2.88 | | | |  |
|  | |  | | | |  | | | |  | | | |  |  | | | | | |  | |  | | | |  | |  | | |  | |  | | | |  | |  | | | | |  | |  | | |  | |  | | |  |  | | | | |  | |  | | | |  | |  | | | |  | |  | |  | |  | |  |  | | | | | |  | | |  | | | |  |
|  | | | **Mito-PEG_5_-ATO (µM)** | | | | | | | | | | | | | | | | | | | | | | | | | | | | | | | | | | | | | | | | | | | | | | | | | | | | | | | | | | | | | | | | | | | | | | | | | | | | | | | | | | | | | | | | | | | |  |
| **AZD-3965 (µM)** | **0** | | | | | | | | | | |  | **0.02** | | | | | | | | | | | |  | | | **0.04** | | | | | | | |  | | | **0.06** | | | | | | | | | |  | | **0.08** | | | | | | | | | | |  | | **0.1** | | | | | | | | | |  | | **0.15** | | | | | | |  | | | **0.5** | | | | | | | | | |
| 0 | 28.65 | | | | ± | | 0.85 | | | | |  | 34.79 | | | | | | | ± | | 0.88 | | |  | | | 29.69 | | | ± | | 1.89 | | |  | | | 29.11 | | | | ± | | | 2.08 | | |  | | 28.15 | | | ± | | | 1.61 | | | | |  | | 27.57 | | | | ± | | 1.60 | | | |  | | 28.14 | | ± | | 2.75 | | |  | | | 26.75 | | | | ± | | | 1.89 | | |
| 10 | 28.98 | | | | ± | | 1.41 | | | | |  | 34.88 | | | | | | | ± | | 1.58 | | |  | | | 30.53 | | | ± | | 2.18 | | |  | | | 28.31 | | | | ± | | | 3.34 | | |  | | 29.16 | | | ± | | | 1.00 | | | | |  | | 28.83 | | | | ± | | 1.89 | | | |  | | 28.55 | | ± | | 1.06 | | |  | | | 27.91 | | | | ± | | | 1.67 | | |
| 20 | 29.42 | | | | ± | | 0.86 | | | | |  | 31.98 | | | | | | | ± | | 3.32 | | |  | | | 29.51 | | | ± | | 1.65 | | |  | | | 29.13 | | | | ± | | | 1.65 | | |  | | 28.90 | | | ± | | | 1.41 | | | | |  | | 29.31 | | | | ± | | 1.28 | | | |  | | 28.96 | | ± | | 1.41 | | |  | | | 27.56 | | | | ± | | | 1.31 | | |
| 30 | 28.90 | | | | ± | | 1.52 | | | | |  | 32.14 | | | | | | | ± | | 1.00 | | |  | | | 29.18 | | | ± | | 1.57 | | |  | | | 29.34 | | | | ± | | | 1.26 | | |  | | 29.69 | | | ± | | | 1.91 | | | | |  | | 29.03 | | | | ± | | 2.46 | | | |  | | 29.04 | | ± | | 1.79 | | |  | | | 27.83 | | | | ± | | | 1.29 | | |
| 40 | 30.34 | | | | ± | | 1.54 | | | | |  | 31.69 | | | | | | | ± | | 1.20 | | |  | | | 29.36 | | | ± | | 0.66 | | |  | | | 30.46 | | | | ± | | | 1.12 | | |  | | 29.08 | | | ± | | | 2.31 | | | | |  | | 29.12 | | | | ± | | 0.72 | | | |  | | 28.89 | | ± | | 1.91 | | |  | | | 28.21 | | | | ± | | | 2.45 | | |
| 60 | 30.77 | | | | ± | | 1.10 | | | | |  | 32.94 | | | | | | | ± | | 2.35 | | |  | | | 30.65 | | | ± | | 0.17 | | |  | | | 31.56 | | | | ± | | | 0.94 | | |  | | 29.57 | | | ± | | | 2.24 | | | | |  | | 30.15 | | | | ± | | 1.16 | | | |  | | 30.18 | | ± | | 1.31 | | |  | | | 30.39 | | | | ± | | | 2.23 | | |
| 80 | 30.40 | | | | ± | | 0.82 | | | | |  | 30.08 | | | | | | | ± | | 5.46 | | |  | | | 29.83 | | | ± | | 0.46 | | |  | | | 29.99 | | | | ± | | | 1.15 | | |  | | 29.91 | | | ± | | | 0.96 | | | | |  | | 31.08 | | | | ± | | 1.86 | | | |  | | 30.34 | | ± | | 1.55 | | |  | | | 29.71 | | | | ± | | | 1.63 | | |
| 100 | 31.41 | | | | ± | | 1.01 | | | | |  | 32.54 | | | | | | | ± | | 2.20 | | |  | | | 30.50 | | | ± | | 2.47 | | |  | | | 31.20 | | | | ± | | | 1.39 | | |  | | 30.06 | | | ± | | | 1.51 | | | | |  | | 30.12 | | | | ± | | 1.22 | | | |  | | 30.09 | | ± | | 2.45 | | |  | | | 30.26 | | | | ± | | | 1.50 | | |
| 150 | 32.36 | | | | ± | | 1.46 | | | | |  | 34.03 | | | | | | | ± | | 2.67 | | |  | | | 31.05 | | | ± | | 0.52 | | |  | | | 31.49 | | | | ± | | | 1.31 | | |  | | 30.74 | | | ± | | | 1.93 | | | | |  | | 30.11 | | | | ± | | 0.92 | | | |  | | 30.74 | | ± | | 0.82 | | |  | | | 30.80 | | | | ± | | | 0.54 | | |
| 200 | 30.51 | | | | ± | | 1.79 | | | | |  | 30.25 | | | | | | | ± | | 2.71 | | |  | | | 29.52 | | | ± | | 1.05 | | |  | | | 30.80 | | | | ± | | | 1.38 | | |  | | 29.02 | | | ± | | | 2.18 | | | | |  | | 28.89 | | | | ± | | 0.96 | | | |  | | 29.22 | | ± | | 1.72 | | |  | | | 28.27 | | | | ± | | | 2.21 | | |
| 400 | 31.60 | | | | ± | | 1.56 | | | | |  | 29.24 | | | | | | | ± | | 2.91 | | |  | | | 31.04 | | | ± | | 0.91 | | |  | | | 31.64 | | | | ± | | | 1.59 | | |  | | 31.08 | | | ± | | | 0.88 | | | | |  | | 30.09 | | | | ± | | 2.35 | | | |  | | 30.31 | | ± | | 2.33 | | |  | | | 29.39 | | | | ± | | | 1.43 | | |
| 600 | 31.63 | | | | ± | | 3.49 | | | | |  | 29.02 | | | | | | | ± | | 1.50 | | |  | | | 30.43 | | | ± | | 2.45 | | |  | | | 30.68 | | | | ± | | | 2.27 | | |  | | 29.29 | | | ± | | | 3.30 | | | | |  | | 28.38 | | | | ± | | 3.41 | | | |  | | 29.23 | | ± | | 1.79 | | |  | | | 28.38 | | | | ± | | | 1.82 | | |
